# Current Trends of Raman Spectroscopy in Clinic Settings: Opportunities and Challenges

**DOI:** 10.1002/advs.202300668

**Published:** 2023-12-10

**Authors:** Yumei Wang, Liuru Fang, Yuhua Wang, Zuzhao Xiong

**Affiliations:** ^1^ Department of Nephrology Union Hospital Tongji Medical College Huazhong University of Science and Technology Wuhan 430022 China; ^2^ Hubei Province Key Laboratory of Systems Science in Metallurgical Process Wuhan University of Science and Technology Wuhan 430081 China

**Keywords:** biomarkers, clinical diagnosis, clinic settings, detection, Raman spectroscopy

## Abstract

Early clinical diagnosis, effective intraoperative guidance, and an accurate prognosis can lead to timely and effective medical treatment. The current conventional clinical methods have several limitations. Therefore, there is a need to develop faster and more reliable clinical detection, treatment, and monitoring methods to enhance their clinical applications. Raman spectroscopy is noninvasive and provides highly specific information about the molecular structure and biochemical composition of analytes in a rapid and accurate manner. It has a wide range of applications in biomedicine, materials, and clinical settings. This review primarily focuses on the application of Raman spectroscopy in clinical medicine. The advantages and limitations of Raman spectroscopy over traditional clinical methods are discussed. In addition, the advantages of combining Raman spectroscopy with machine learning, nanoparticles, and probes are demonstrated, thereby extending its applicability to different clinical phases. Examples of the clinical applications of Raman spectroscopy over the last 3 years are also integrated. Finally, various prospective approaches based on Raman spectroscopy in clinical studies are surveyed, and current challenges are discussed.

## Graphical abstract

The paper systematically demonstrates the status and trends in the application development of Raman combined with machine learning, nanoparticles, and probes in typical clinical diseases. The focus is to explore the limitations and sources of errors in traditional techniques, providing a comprehensive showcase of the typical research outcomes of Raman in clinical settings over the past 3 years. This review offers profound insights for future research.

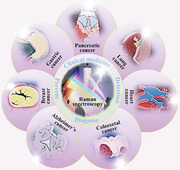

## 1 Introduction

Nearly 75% of all deaths worldwide are caused by chronic illnesses such as cancer, heart disease, and lung disease, which have significantly increased in prevalence.^[^ ^1^ ^]^ The prompt and efficient administration of treatments is facilitated by an early clinical diagnosis, effective intraoperative guidance, and reliable prognosis. Currently, diagnostic techniques incorporating magnetic resonance imaging (MRI), computed tomography (CT), and sonography are heavily relied upon by doctors. Although some of these methods can image deep tissues, they have poor spatial resolution and offer only a limited understanding of the biochemistry underlying the disease.^[^ ^2^ ^]^ The need for expensive high‐tech tools and the negative effects of employing ionizing radiation are two additional disadvantages. Furthermore, histopathological recognition is still a time‐consuming, subjective procedure hampered by interobserver variability, despite its importance for clinical and research activities.

**Figure 1:**
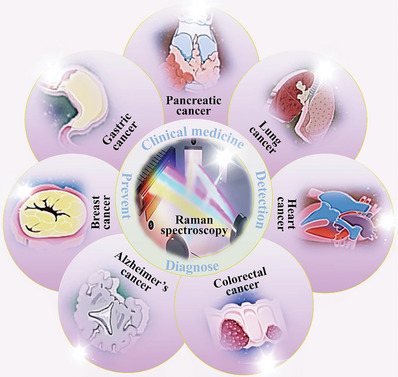
Application of Raman spectroscopy in clinical medicine, with respect to various common diseases.

Therefore, to overcome these problems and achieve effective early diagnosis, there is an urgent need to introduce tools that are non‐invasive, dependable, quick, affordable, and can detect the disease at an early manageable stage, monitor the effects of treatment, and help guide interventions. Raman spectroscopy meets these requirements and has been used in a wide range of clinical applications since its discovery (**Figure** **1**). In light of these inherent benefits and its ability to provide a thorough chemical profile of biological materials, Raman spectroscopy provides a quick and inexpensive molecular fingerprinting platform with the potential to create novel detection pathways in therapeutic situations.^[^ ^3^, ^4^, ^5^ ^]^

Furthermore, we observed in a Web of Science citation report that, based on a search for publications related to “Raman spectroscopy,” “medical diagnosis,” and “medical treatment” between 2000 and 2023, the annual citation count gradually increased from 306 to 13117. This special issue focuses on advancing the fundamental aspects of bringing this field closer to clinical application. Given the rapid evolution within this domain, it is essential to periodically summarize the latest developments in the clinical application of Raman spectroscopy, address the current challenges, and envision future trends.

This review presents a comprehensive heuristic summary of the use of Raman spectroscopy in clinical practice. Furthermore, an objective analysis of the current challenges of Raman spectroscopy in clinical applications was conducted. This includes an impartial assessment of issues such as technical limitations, challenges in data interpretation, sources of errors, and related matters, along with the validation of the advantages of surface‐enhanced Raman spectroscopy (SERS). In addition, the current problems of Raman spectroscopy in clinical applications have been analyzed objectively. The combination of Raman spectroscopy with machine learning, nanoparticles (NPs), and probes overcomes these limitations and demonstrates the advantages of these new applications. Owing to rapid advancements in the field of medicine in recent years, there has been an accelerated trend in the innovation and progress of Raman spectroscopy in clinical applications. To ensure that the review encompasses the most up‐to‐date developments and trends, we summarized a significant portion of Raman spectroscopy research in clinical trials over the past 3 years. Additionally, we provide detailed insights into breast and prostate cancer (PCa), which have been extensively studied during these 3 years. Based on these two common diseases, we analyzed the current status of Raman spectroscopy in clinical applications. Finally, we present a comprehensive and systematic summary of Raman spectroscopy in a clinical setting, discussing the ongoing controversies in the field, current trends, and future directions. We hope that this review will arouse interest in this field and that new contributions can be made to future research.

## 2 Raman Spectroscopy

### 2.1 Principles of Raman Spectroscopy

Raman spectroscopy was used to study materials based on the Raman effect, which is the inelastic scattering of incoming light of a material and its frequency changes due to the power of excited molecular vibrations. Stokes scattering can result in a frequency redshift when it begins from the vibrational ground state; however, when it begins from an excited vibrational state, the incident photon may acquire energy when dispersed (anti‐Stokes scattering).^[^ ^6^, ^7^ ^]^ Because each fingerprint possesses unique variations, the generated Raman spectrum is closely linked to the molecular structure and vibrational characteristics of different materials, exhibiting a high degree of individualization and scientific distinctiveness. Generally, Raman spectroscopy can detect rotational or other low‐frequency modes; however, these have no bearing on the clinical applications discussed herein. In the context of Raman spectroscopy, “low‐frequency modes” typically refer to vibrational modes of molecules that involve large‐scale or collective motions, such as molecular rotations and translational movements of entire molecular segments. These modes have lower frequencies than high‐frequency vibrational modes, which involve localized atomic vibrations within the molecule.

The molecules that constitute the cells and tissues of interest vibrate in ways that induce distinct variations in the energy of dispersed sunlight. This method can be used noninvasively because it uses light to examine disease‐specific molecular alterations, especially in situations where fiber‐optic access is feasible. The gathered molecular data can be used to distinguish between different cell and tissue types as well as between diseased and healthy tissues. Raman spectroscopy‐based instruments can provide clinical tools that are precise and little‐to‐non‐invasive and have distinct capabilities that are comparable to or even better than gold standard methods. Different chemical bonds and functional groups generate specific spectral signals that can be used to identify the type and structure of the molecules. Raman spectroscopy can detect these vibrational changes in molecules and provide insight into their molecular composition and structure within cells. By comparing the Raman spectra of normal cells and cells affected by epigenetic changes, researchers can identify the molecular changes associated with diseases. These changes may involve variations in the content or structure of biomolecules such as proteins, nucleic acids (NAs), lipids, and other biological molecules, offering significant insights into molecular disease research.^[^ ^8^, ^9^ ^]^

### 2.2 Different Types of Raman Spectroscopy

Raman spectroscopy provides a non‐destructive and high‐throughput evaluation of the results. Moreover, it is relatively inexpensive and requires minimal background training, thus exhibiting significant potential for clinical applications.^[^ ^10^, ^11^ ^]^ However, the intensity of the Raman scattering signal is low, and it exhibits a low anti‐interference capacity. Therefore, a long time duration is required to obtain an acceptable signal‐to‐noise ratio (SNR) for practical applications, and spectral images cannot be captured rapidly in clinical applications. In addition, most biological samples tested in clinical practice have a high background fluorescence, which further reduces the SNR of the Raman spectrum. To solve this problem, various Raman signal enhancement methods have been studied, of which SERS is the most commonly used. In Section 2.5, SERS will be covered in detail. There are several types of Raman spectroscopy, including needle‐tip Raman spectroscopy (TERS)^[^ ^16^ ^]^ and stimulated Raman spectroscopy (SRS). These types of Raman spectroscopy have been used in clinical diagnosis and treatment to various extents. These technologies exhibit high biochemical sensitivities and selectivities. **Table** **1** lists the advantages and limitations of different types of Raman spectroscopy.

**Table 1:** Different types of commonly used Raman spectroscopy.^[^ ^10^, ^11^, ^12^, ^13^, ^14^, ^15^ ^]^

Types of Raman Spectroscopy	Benefits	Limitations	Clinical Applications
Raman Spectroscopy 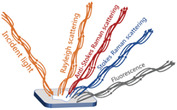	The system is simple. General backscatter. Confocal operations allow for narrow 3D imaging. Wider than infrared absorption. Small sample proportion.	Low sensitivity. Susceptible to scattering, fluorescence, and instrument calibration drift. Requires point‐to‐point mapping. The process is time‐consuming.	Analyze biomolecules such as proteins, nucleic acids (NAs), and lipids.
Surface Enhanced Raman Spectroscopy (SERS) 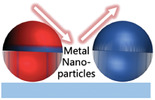	Signal enhancement. Slight external influence. Clearer than the infrared spectrum. Substrates or colloids are generally used for suspension.	The spectrum of endogenous molecules could change. Poor reproducibility. Samples require pre‐treatment. Selective for adsorbed detection molecules.	Detection of trace biomolecules such as cancer markers.
Resonance Raman Spectroscopy (RRS) 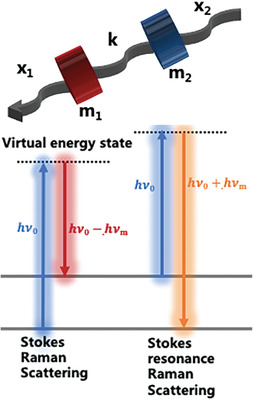	Compared with traditional Raman spectroscopy, the signal is enhanced, and the imaging speed is increased.	A readily adjustable laser with a continuous frequency is required.	Analyzing microstructural changes in biomolecules.
Tip‐enhanced Raman Spectroscopy (TERS) 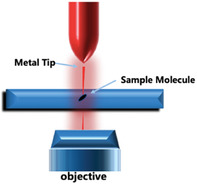	Low spatial resolution. It exhibits superior spectral characteristics to the infrared spectrum. Increased anti‐fluorescence interference performance.	The detection process is more complex. Poor reproducibility and tip heating.	Molecular structures can be observed on the cell surface.
Coherent Anti‐Stokes Raman Scattering (CARS) 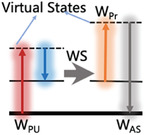	Low spatial resolution. Demonstrates superior spectral characteristics to the infrared spectrum. Increased anti‐fluorescence interference performance.	Equipment is expensive. The non‐resonant background is strong and may lead to the photothermal damage of the sample.	Real‐time imaging can be realized for observing the movement and distribution of molecules in living cells.
Stimulated Raman Spectroscopy (SRS) 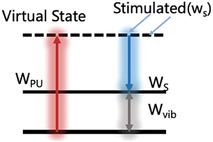	Signal enhancement. High imaging speed. It is not influenced by non‐resonant backgrounds and fluorescence. High sensitivity.	Equipment is expensive. Samples are at risk of photothermal damage.	Increased signal intensity is suitable for dynamic monitoring of intracellular molecular responses.
Spatially Offset Raman Spectroscopy (SORS) 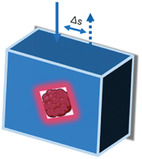	Enhanced anti‐fluorescence interference ability. Can provide deep organizational information.	Data processing is complex. Raman signal is relatively weak. Poor reproducibility.	Suitable for analyzing the chemical composition of tissue samples.
Surface‐Enhanced Spatially Offset Raman Spectroscopy (SESORS) 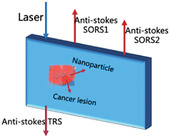	The detection depth can reach 50 mm below the surface.	NPs are required as a medium.	Trace molecules can be detected in deep tissues.
Shifted‐Excitation Raman Difference Spectroscopy (SERDS) 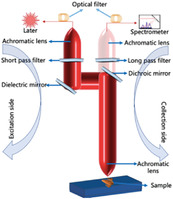	It has a Raman fluorescence rejection reaction.	Raman difference spectra are reconstructed by fitting peaks.	The effect of light scattering inside the sample can be eliminated, improving the SNR.

### 2.3 Recent Experimental Studies of Different Raman Spectroscopy

The zero‐offset diffuse beam used to detect lesions at the sampling volume (surface‐to‐epidermal‐to‐dermal junction) can provide an optimal SNR,^[^ ^17^ ^]^ which serves as a basis for future Raman spectroscopy in skin cancer detection. In skin cancer detection, spatially offset Raman spectroscopy (SORS) can collect Raman signals from deep layers of the skin tissue, aid in the identification of lesions, and provide more accurate diagnostic information. Similarly, in bone tissue regeneration monitoring, SORS can be employed to measure changes in collagen protein concentration within deep bone tissue, thereby enabling the assessment of the skeletal health status.^[^ ^18^ ^]^ Dooley et al.^[^ ^19^, ^20^ ^]^ investigated the feasibility of using SORS to monitor collagen concentrations (0–0.84 g cm^−3^) during bone tissue regeneration.

Li et al.^[^ ^21^ ^]^ used a coherent anti‐Stokes Raman microscope to image cells. The analysis of numerous biological phenomena involving lipid metabolism, cancer formation, cardiovascular disease, and skin biology has been made possible by the connection between two‐photon fluorescence, second‐harmonic generation, and coherent anti‐Stokes Raman scattering (CARS).

Surface‐enhanced spatial offset Raman spectroscopy (SESORS) combines the signal enhancement provided by SERS with the diffusion scattering provided by SORS to obtain deeper signals in vivo than conventional Raman instruments. Berry et al.^[^ ^22^ ^]^ first demonstrated that SESORS signals could be detected in nanotags up to a depth of 48 mm. CARS imaging has been extensively conducted for molecular vibration imaging of cells and tissues. CARS operates by utilizing the coherent interactions between laser beams and molecular vibrations, resulting in the generation of a unique signal that can be used to create detailed chemical images of samples without the need for exogenous labels. They have found significant applications in the study of biological systems and material analysis. Mizuguchi et al. made a significant discovery regarding the utility of CARS imaging, which enables visualization of the entire intracellular lipid structure. Additionally, this unique capability can be harnessed for real‐time tracking of living cells with intracellular lipid structures.^[^ ^23^ ^]^ Building on this foundation, Fueser et al. conducted comprehensive investigations employing CARS microscopy on Caenorhabditis elegans, thereby illustrating the suitability of this imaging technique for single‐organism imaging.^[^ ^24^, ^25^ ^]^ These studies highlight the substantial potential of CARS microscopy in clinical diagnostics. Furthermore, Kaneta et al. employed an advanced technique known as ultra‐multiplex CARS microscopy to meticulously examine the distribution of water concentration in human skin in vitro.^[^ ^26^ ^]^ This innovative approach allows for the detection of potential pathological alterations arising from improper water distribution within the human skin. Collectively, these research endeavors emphasize the versatility and clinical relevance of CARS microscopy in diverse scientific domains.

SRS microscopy, in combination with second‐harmonic generation (SHG) microscopy, has been used to visualize tissue sections within the human gastrointestinal tract from both healthy individuals and cancer patients. In recent years, rapid advancements in machine learning and artificial intelligence have led to remarkable progress in image‐based medical diagnostics. Several research groups have successfully developed SRS histology methods integrated with deep learning algorithms to enable the swift diagnosis of various human tumors, including brain tumors and laryngeal cancers.^[^ ^27^, ^28^, ^29^ ^]^ For instance, Zhang et al. employed a coherent nonlinear optical microscopy technique that combined SRS with SHG to conduct label‐free histology of the human pancreatic tissue. Furthermore, they explored the potential of machine and deep learning for automating the identification and analysis of crucial histological features.^[^ ^30^ ^]^ The significance of these studies extends beyond technological advancements in the domain of medical image diagnostics. They also provide clinicians with expedited and more precise diagnostic tools, which are particularly pivotal in the early detection and treatment of ailments such as cancer. Further studies are required to validate the feasibility of these methods and expand their application in clinical practice.

### 2.4 Raman Spectrometers

The spectrometer, detector, and excitation laser are the three main parts of a Raman instrument.^[^ ^31^ ^]^ The spectrometer serves as the central element within the Raman instrument and is responsible for the separation and measurement of distinct wavelength components inherent in the light scattered by the sample. This task is achieved through the utilization of optical elements, such as gratings or interferometers, to attain spectral resolution and measurement precision. However, the detector plays a crucial role in capturing segregated spectral signals. It effectively converts these signals into their electrical counterparts, which are subsequently subjected to comprehensive analysis and processing. An excitation laser is a pivotal component in the generation of an excitation light source. These lasers can be of various types, including laser diodes, gas lasers, or solid–state lasers. Each variation in the excitation light source possesses unique wavelength and power attributes, enabling tailored selection based on the specific requirements of the sample at hand. The sample is stimulated by transmitting an excitation light source through an optical fiber, causing its molecules to undergo Raman scattering.

Moreover, a clinical Raman system requires optical alignment and calibration, sample light delivery and collection, durability and sterility, and detection system sensitivity. In addition, the device must be small and portable. These features are similar to those of standard medical instruments. The excitation and detection branches can be used to broadly group the parts that constitute a clinical Raman system. Excitation occurs when a fiber‐optic probe or articulated light delivery arm transmits light from a particular laser source to a target tissue spot. The gathered Raman‐scattered light is then sent to a spectrograph and detector using a common delivery method. The development and implementation of clinical Raman systems present a range of complex challenges. First, achieving precise alignment and calibration of the optical pathway is pivotal for ensuring consistent and accurate measurements. Second, the intricacies of practical operations in a genuine clinical environment must be considered when designing optical delivery and collection mechanisms for samples. Moreover, given that clinical scenarios often require prolonged instrument usage, the apparatus must be robust and sterile to guarantee reliability and safety. One of the defining merits of clinical Raman systems is their non‐invasive nature. Leveraging fiber‐optic conduits or light‐guided arms, light can be seamlessly directed into target tissues without necessitating incisions or sampling procedures. This not only minimizes patient discomfort and risk but also facilitates real‐time monitoring. Furthermore, Raman spectroscopy provides insights intrinsically tied to the chemical composition, thus fostering applications ranging from disease diagnostics and drug surveillance to advanced biological research. The range of individual components that have been researched and approved for use in these systems has expanded along with the technology used in vivo for clinical assessments.

### 2.5 Raman Spectroscopy for Signal Enhancement

SERS, initially used for the identification of singular molecules, has undergone transformative evolution, emerging as one of the most versatile modalities for the sensitive detection and intricate visualization of chemical and biological analytes.^[^ ^32^ ^]^ SERS primarily involves physical and chemical enhancement mechanisms. Physical enhancement relies mainly on nanostructures made of metals such as Ag and Au. These structures exhibit relative stability, a high degree of repeatability, and ease of preparation. They can induce collective oscillations of free electrons on the surface, significantly enhancing the intensity of Raman signals.^[^ ^33^, ^34^ ^]^ The latter is used to enhance the Raman signal via a charge‐transfer mechanism, which requires the examined materials to be adhered or glued directly to a rough metal substrate. These materials can undergo charge transfer to the metal surface to enhance the Raman signal. Moreover, SERS can be used to analyze a substance, and its concentration and location can be determined by measuring its Raman strength.^[^ ^35^, ^36^, ^37^, ^38^ ^]^ Moreover, this technique has the potential to ascertain the spatial distribution of the analytes. Advancements in SERS have made it a pivotal tool in molecular analyses. The unique synergy between nanostructures, charge transfer, and precise Raman measurements presents a sophisticated platform for both the qualitative and quantitative assessment of various analytes, thereby contributing substantially to analytical chemistry, biomedicine, and materials science.

**Figure 2:**
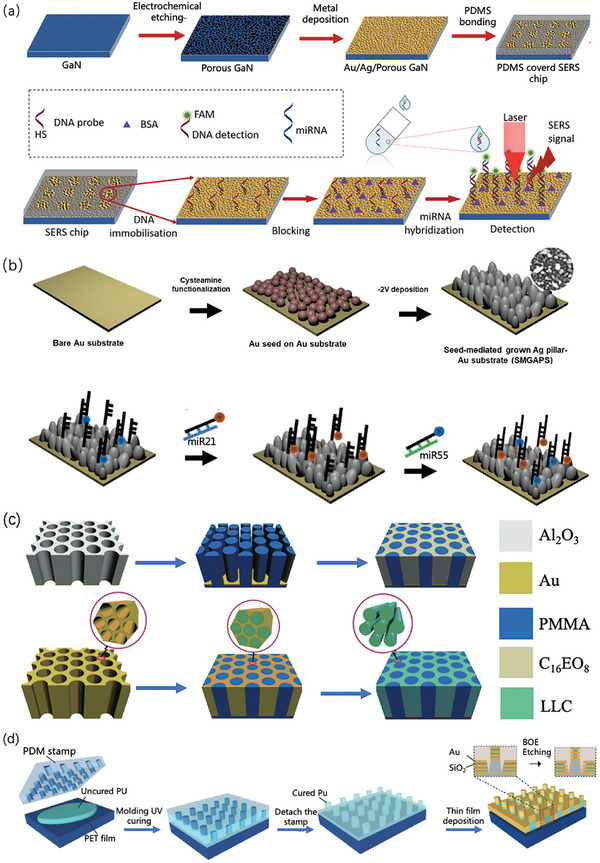
Schematics of different enhancement methods for Raman signal. a) Schematic of the preparation principle of SERS with Au or Ag hybrids based on porous GaN and the utilization of the method for miRNA detection.^[^ ^138^ ^]^ Copyright 2023, Elsevier. b) Seed‐mediated growth of Ag pillar and the utilization of the method for miRNA detection.^[^ ^40^ ^]^ Copyright 2023, Elsevier. c) 3D schematic of SERS substrate preparation.^[^ ^41^ ^]^ Copyright 2023, John Wiley and Sons. d) The preparation process of SERS with alternating metal–insulator–metal layer.^[^ ^42^ ^]^ Copyright 2023, John Wiley and Sons.

As depicted in **Figure** **2**a, GaN wafers were etched.^[^ ^39^, ^138^ ^]^ Thereafter, a Ag layer was deposited on the substrate, and a thin layer of Au was deposited. As demonstrated, the SERS substrate prepared using this method can detect microRNA (miRNA), which is a biomarker of breast cancer. Kim et al.^[^ ^40^ ^]^ reported a seed‐mediated growth method for Ag pillar SERS substrates in 2022. Silver ion development was supported by gold nanoparticle seeds (sGNP) that were electrochemically reduced to a size of 40 nm; on the gold substrate surface, a silver structure in the form of nanopillars was effectively produced. Within the synthesized structure, the malachite green isothiocyanate signal was significantly enhanced. Figure 2b shows a schematic of miRNA detection using the seed‐mediated silver column growth method.

Metal nanostructures have emerged as crucial elements in the field of plasma antennas, contributing to the augmentation of Raman signals in SERS‐based molecular detection. Evidently, the Raman signal can be significantly intensified within specific nanoenvironments such as nanopores, ion bond gaps, or nanotips. Zhang et al.^[^ ^41^ ^]^ investigated the intricate fabrication of porous metamaterials at the 3D level by leveraging dual‐template technology. This approach provides a finer degree of control over the structural attributes of the plasma employed for SERS. In their study, the dual‐templating methodology incorporated a firm template composed of porous poly (methyl methacrylate) (PMMA) to fabricate hierarchically ordered porous metamaterials in a 3D configuration. Lyotropic liquid crystals (LLCs) derived from octaethylene glycol monohexadecyl ether (C16EO8) served as precursors for this dual‐templating strategy. Notably, the SERS substrates encompass various architectural compositions: periodic arrays of nanoholes, or NPs; newly devised nanoporous structures; and individual NPs characterized by distinct geometric attributes, as depicted in Figure 2c. Despite the diversity of their structures, all three SERS substrate types possessed single‐scale architectures.

This exploration of nanostructured plasma antennas, porous metamaterials, and diverse substrate architectures advances our understanding of the enhanced Raman signal generation within the SERS framework. This underscores the intricate interplay between nanostructures, plasma interactions, and material engineering, ultimately enhancing the sensitivity and precision of molecular detection applications.

Song et al.^[^ ^42^ ^]^ investigated a top‐down preparation method for preparing high‐performance SERS substrates. Scalable nanolaminated SERS substrates were fabricated using a top‐down technique, as shown in Figure 2d. Song et al. initially created replicas of polymer nanopillar arrays on polyester (PET) films using a UV‐curable device with nanostructured polydimethylsiloxane (PDMS) stamp and a UV‐curable polyurethane (PU) polymer resist, and polymer nanopillar arrays were created on polyester (PET) films. The construction of multilayered MIM nanostructures with multiple vertically oriented plasmonic nanogaps on individual nanopillars was followed by the alternating deposition of layers of Au and SiO_2_ films. Finally, to enhance the performance of SERS, they exposed the embedded nanogap hotspots using a wet chemical etching method and a buffered oxide etchant (10:1) (BOE). This strategic manipulation of the nanogaps and plasmonic elements enhances SERS efficiency, thereby rendering the substrate more sensitive and effective in detecting target analytes. This comprehensive approach showcases the systematic fabrication of nanolaminated SERS substrates marked by intricate nanopillar arrays and precisely engineered plasmonic nanogaps. The collective efforts to design, fabricate, and optimize these structures underscore the importance of harnessing the full potential of SERS substrates for enhanced analytical capabilities.

**Figure 3:**
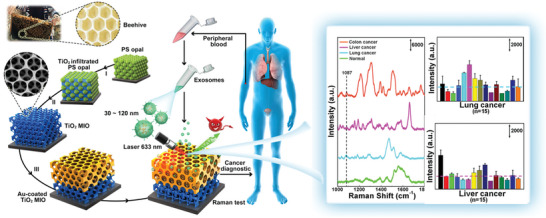
Structure and action process of TiO2 microporous reverse opal coated with 3D Au. The interaction with the laser sample in SERS enhanced the Raman signal.^[^ ^37^ ^]^ Copyright 2020, American Chemical Society.

Additionally, an engineered “slow light effect” on a macroporous TiO_2_ inverse opal (MIO) structure inspired by beehives and exceptional SERS performance was created by Dong et al.^[^ ^37^ ^]^ Without the use of labeling procedures, the MIO structure collects and examines exosomes from the cancer patients’ plasma.^[^ ^43^ ^]^ **Figure** **3** presents a schematic of this process, and the results demonstrate improved efficiency and accuracy in the detection of multiple cancers. Exosomes from the plasma's 1087 cm^−1^ SERS peak were used in this study to provide a label‐free, adaptable, inexpensive, and quick method for obtaining reliable tumor liquid biopsy. This method has considerable clinical potential for use in rapid in vitro cancer screening. Dong's interesting thought process also provides insights into prospective developments.

SERS enhances the sensitive excitation and scattering of plasmons while retaining abundant chemical fingerprint information from Raman spectroscopy. In particular, the measurements can be performed conveniently under ambient and aqueous conditions, and the narrow width of the majority of Raman peaks makes them suitable for multiplex analyses. SERS is well recognized as a reliable analytical method for identifying the molecular signatures of potentially interesting analytes for clinical translation.^[^ ^44^ ^]^ In several biomedical applications, particularly in disease diagnosis, significant progress has been made in the production of highly sensitive SERS substrates. However, the effect of fouling on substrates with SERS activity has severely hampered their direct clinical application in biological fluids. This is also a challenge that must be addressed to achieve the clinical conversion of SERS in the future.

## 3 Clinical Applications of Raman Spectroscopy

Currently, cytological assessments, immunohistochemistry, and imaging techniques are commonly used in clinical trials. However, these methods are time‐consuming, expensive, and lack automation. Histopathological testing is extensively used in cancer diagnosis and can be considered the standard test procedure, although it has several limitations. Generally, cancer detection relies primarily on the evaluation of microscopic sample staining by pathologists.^[^ ^101^ ^]^ However, this method is invasive, time‐consuming, and has limited sensitivity because it uses dyes that are not specific to cancer cells. Second, the number of pathologists who could conduct professional evaluations is limited. Additionally, this method is overly dependent on the subjective judgment of pathologists and lacks objectivity. Therefore, misdiagnosis is common during tissue evaluations. Improved clinical diagnostic methods are required to overcome these limitations and meet the clinical requirements.

Raman spectroscopy is sensitive to the biochemical signs of diseases in a variety of medical samples. Owing to the adaptability of these vibrational approaches, customized analyses, which may include signature‐based strategies, can be designed based on clinical needs to try and grasp the complexity of illness profiles for diagnosis or to identify a particular and extremely sensitive target molecule. Most vibrational spectroscopic techniques are straightforward, reliable, and depend on simple sample preparation methods. Additionally, the gear can be tailored for specific applications (e.g., probe‐based, handheld, and imaging).^[^ ^102^ ^]^ These benefits make them medically useful and appropriate for clinical settings, owing to their capacity for the highly sensitive and specific distinction of diseased from non‐diseased states and disease severity in certain situations. The application of Raman spectroscopy to clinical issues has advanced rapidly in recent years, demonstrating the capability of spectroscopy to offer objective data to support clinical decision‐making and expand our fundamental knowledge of how light interacts with matter.

### 3.1 Multivariate Analysis Tools

Several statistical techniques, including supervised and unsupervised learning techniques, can be employed for clinical sample categorization because the SERS spectra can be viewed as multivariant data.^[^ ^45^, ^46^ ^]^ Unsupervised learning techniques address and identify the structures and patterns in data with no outcomes. Principal component analysis (PCA) and hierarchical cluster analysis (HCA) are two techniques widely used in SERS spectral analysis. Similar spectra can be grouped using cluster analysis, even if the labels are unknown. Supervised learning techniques can be divided into classification and regression methods, depending on the type of output: label or continuous value. SERS spectral differentiation has been extensively achieved using supervised learning techniques such as partial least squares discriminant analysis (PLS‐DA), partial least squares regression (PLS), linear discriminant analysis (LDA), support vector machines (SVM), k‐nearest neighbor (KNN), random forest (RF), and convolutional neural networks (CNNs). This is particularly true for distinguishing the SERS spectra of analytes in complex biological matrices.^[^ ^47^ ^]^

**Figure 4:**
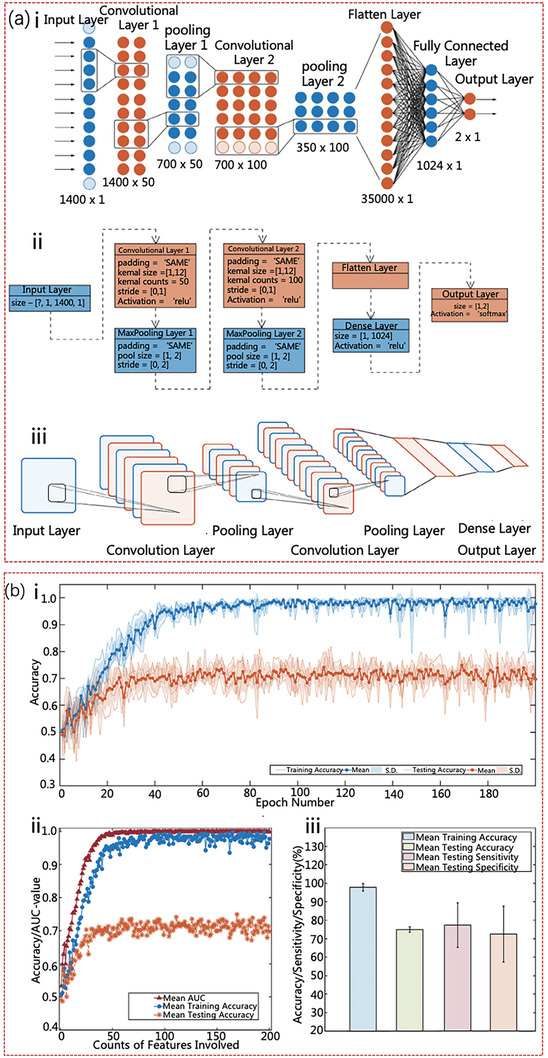
Schematic and parameter representation of the improved LENET‐5 model and results of training and testing using the model. a) Improved mode and calculation. b) Quintuple crossover and verification results of the CNN model in the improved Lenet‐5 Raman spectrum processing model.^[^ ^48^ ^]^ Copyright 2021, Wiley‐VCH.

Chen et al. integrated Raman spectroscopy with CNNs to introduce a novel approach for detecting PCa through urine analysis.^[^ ^48^ ^]^ PCa and prostatic hyperplasia (BPH) are discernible through variations in the intensity of the characteristic peaks related to lipids, NAs, and selected amino acids within the urine Raman spectra. This differential pattern indicates the presence of anomalous metabolic activity associated with PCa, which can be captured by Raman spectroscopy. Subsequently, these data are used to train a CNN‐based intelligent diagnostic model. According to the cross‐validation results, the average diagnostic accuracies for Pca were 74.95%, 77.32%, and 72.46%, respectively. This synergistic application of artificial intelligence augmented the efficacy of Raman spectroscopy for PCa detection. Notably, the nodes indicated near the base of (i) signify the actual nodes within the model, whereas (ii) denote the nodes within the improved Lenet‐5 model. A schematic representation of the CNN model in the improved Lenet‐5 configuration is presented in (iii) and illustrated in **Figure** **4**a. Because of the multitude of nodes, only a partial representation of the underlying nodes in (i) is depicted in the figure, and (ii) encapsulates the node count and parameter configurations for each layer within the enhanced Lenet‐5 Raman spectrum‐processing model. In Figure 4b, the iterative procedure of the CNN model is delineated; the blue segments correspond to training, the orange segments denote testing, and the translucent region signifies the standard deviation. In addition, (ii) illustrates the evolution of the model's average predictive performance with an increase in the number of epoch elements, and (iii) presents the mean sensitivity and specificity of the training and test accuracies across the 169 epochs. Furthermore, Chen et al. conducted innovative clinical investigations on PCa utilizing artificial intelligence techniques.^[^ ^49^ ^]^ Notably, the analysis of urine samples using Raman spectroscopy revealed discernible variations in the intensity of distinctive peaks associated with lipids, NAs, and selected amino acids, enabling differentiation between Pca and BPH. This observation underscores the potential of Pca to induce the aberrant metabolic shifts manifested in Raman spectroscopy readings. CNN methodology was subsequently employed to construct an intelligent diagnostic model using these datasets.

**Figure 5:**
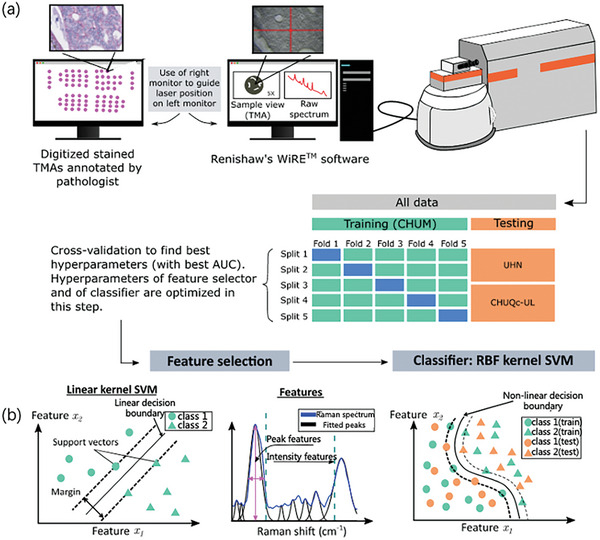
Schematic of data acquisition and machine learning. a) Working flow diagram after combining Raman spectroscopy with machine learning, in which staining TMAs can be used to guide the process of Raman microscope spectral measurement. b) The orange part represents the predicted results in the test set.^[^ ^50^ ^]^ Copyright 2021, Journal of Biomedical Optics.

Similar to the aforementioned study, Plante et al.^[^ ^50^ ^]^ successfully improved machine learning models through dimensionality reduction and classified prostate tissues. The University Health Network (UHN) tested a radial basis function (RBF) kernel support vector machine (SVM) model on two separate cohorts of 76 patients after training it on Raman spectroscopy of prostate tissue from a cohort of 272 patients (Centre Hospitalier de l'Université de Montréal, CHUM) and 135 patients (Centre Hospitalier Universitaire de Québec‐Université Laval, CHUQc‐UL). Both testing sets showed improvement in the area under the curve (AUC).^[^ ^51^ ^]^ **Figure** **5**a illustrates a schematic of the pipeline for data collection and machine learning, in which tissue microarrays (TMAs) are used to guide the Raman microscopy measurements, and a double‐nested quintuple CV is used in the training and test datasets to determine hyperparameters using a random network. The green part in Figure 5b shows the model established using the highest hyperparameter set on the training data, which can be evaluated using the test results (orange part). This method successfully improved classification performance and significantly increased detection efficiency.

Bone metastasis is relatively common in prostate treatment.^[^ ^52^ ^]^ Hu et al.^[^ ^53^ ^]^ developed a PLS‐DA model to classify patients using known radiation toxicity scores and evaluated the degree of bone metastasis of the prostate by evaluating rolling machine neural networks combined with Raman data screening, where SERS combined with a CNN model successfully identified PCa patients with myelitis. The results showed a sensitivity of 95%, a specificity of 92%, and an overall accuracy of 93%. SERS can be used to analyze PCa in blood to predict prostate recurrence. In addition, it is necessary to identify patients at risk of radiation toxicity in clinical practice.^[^ ^54^ ^]^ Patients with radiation toxicity were assessed and classified using Raman spectroscopy. Blood cultures were used in previous experiments to establish a radiation‐sensitive cutoff value of 132, which allows for the personalization of radiation therapy in clinical settings. Moreover, Hu et al.^[^ ^55^ ^]^ successfully used Raman spectroscopy to establish a model for serum testing to predict the sensitivity of patients with PCa to docetaxel chemotherapy, which further provided technical support for chemotherapy.

The above are examples of the successful use of Raman spectroscopy in combination with various models in principal component analysis, linear discriminant analysis (PCA‐LDA), CNN, and PLS‐DA model (all for predictive analysis) examples of machine learning techniques. The results stated above suggest that prostate liquid biopsy using a noninvasive method based on urine Raman spectroscopy and a deep learning algorithm may serve as a model for the use of Raman spectroscopy in research using artificial intelligence in clinical medicine.

### 3.2 Combination with Fiber and Nanoparticles (NPs)

Tissue lesions are complex processes that are accompanied by changes in the content and structure of biomacromolecules. Raman spectroscopy can be used to diagnose analytical materials at the cellular level with greater precision than traditional tissue imaging.^[^ ^56^, ^57^, ^58^ ^]^ However, the detection of exfoliated cells is a non‐invasive method suitable for clinical patients.^[^ ^59^, ^60^ ^]^ Technological advancements in imaging have significantly influenced the development of medical diagnoses and treatments. Raman imaging uses the interaction of light with a material under analysis to examine its molecular structure, geometry, and dynamic characteristics. Raman spectroscopy was combined with optical fiber probes and used for clinical convenience and rapid access to different parts of the patient. However, based on the available literature, no direct use of optical fiber probes for real‐time image acquisition has been reported.

**Figure 6:**
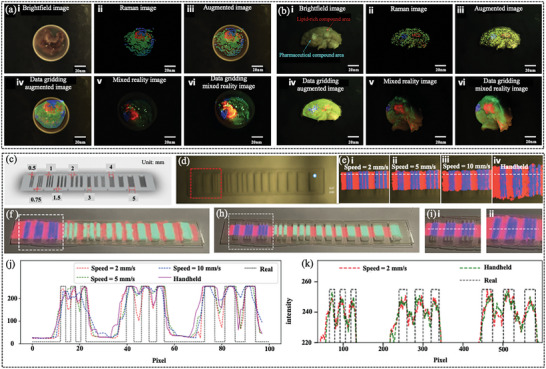
a) In vitro clinical sample testing using this method. b) The method is used to detect pig brain samples. c) Different widths of the stripe space resolution and spatial resolution. d) Objectives of the bright field images and ellipse fitting of laser spot indicator display position information, where the red line is used for imaging the ROI. e) Raman‐enhanced images were displayed by moving the probe at different speeds with five digits, representing white line contour analysis. f,h) Under a speed of 2 mm s^−1^ and the diameter of the reconstructed molecular images combined with the actual Raman image, the white line. i) Details of the ROI. j) The blue path represents Raman‐enhanced images, the black fill represents the paracetamol's actual area, and fill in the blank with paracetamol k) indicated in black.^[^ ^61^ ^]^ Copyright 2022, Springer Nature.

Yang et al.^[^ ^61^ ^]^ examined a Raman imaging system based on a fiber‐optic probe that displayed actual data using virtual molecules on a computer screen in real‐time. **Figure** **6** presents the imaging study and resolution test of this method, which confirms that it is highly suitable for the definition of tissue boundaries in clinical practice as it can rapidly display molecular edges in the distribution of drug compounds and characterize various types of sarcomas. Specifically, (i) presents a bright‐field image, which exhibits difficulty in distinguishing between different molecular regions; (ii) reveals that Raman imaging can readily visualize the positions of different molecules; (iii) reveals that sample information can be distinguished using molecular information enhanced by bright field; (iv) superimposes the data network displaying molecular information and bright field image; (v) presents a visualization of molecular information realized by combining Raman imaging and projection technology; and (vi) presents a reconstructed image of clear molecular boundaries, where green represents epithelial tissue, red represents collagen, and blue represents the scaffold of the plastic sample (Figure 6a).

In Figure 6b, (i) presents the bright‐field image and the area indicated by the arrow is rich in lipids and drug compounds; (ii) displays the reconstructed Raman image of the sample molecular distribution; (iii) fuses the molecular information and bright‐field scene in the actual image to distinguish different regions; (iv) presents the molecular data of the Raman imaging network to form 3D enhanced molecular images; (v) presents the improved Raman image projected onto the sample to reflect clear molecular boundaries; and (vi) uses network data to better display molecular boundaries, where green indicates brain gray matter, blue indicates drug compounds, and red indicates lipid‐rich compounds. The remainder of Figure 6 presents the spatial resolutions of (a) and (b).

This study directly integrates a data‐processing engine into the acquisition flow to address the current drawbacks of conventional Raman systems. This allows us to evaluate the Raman signatures of complex biochemical macromolecules in real‐time and visualize molecules in molecular virtual reality, also known as augmented reality or mixed reality.

The optical detection device for Raman spectroscopy was built into a commercial biopsy machine, enabling clinical analysis before tissue removal without interfering with the workflow. Raman imaging technology has been used in combination with fiber‐optic probes for in situ tissue imaging for various applications.^[^ ^62^, ^63^ ^]^ With the continuous development of optical technology, a combination of optical fiber technology and signal transmission systems can be used to diagnose numerous diseases in clinical practice.^[^ ^64^, ^65^, ^66^, ^67^, ^68^, ^69^, ^70^, ^71^, ^72^ ^]^ In contrast to in vitro detection, in vivo imaging diagnosis using an optical fiber probe is more difficult and less safe; however, when employed, it can significantly promote clinical diagnosis.

**Figure 7:**
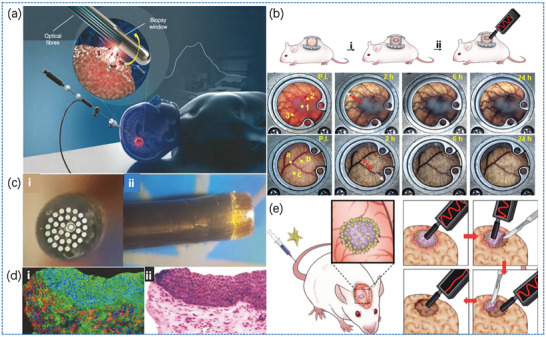
a) Schematic of the optical core probe.^[^ ^73^ ^]^ Copyright 2018, Springer Nature. b) Inset of tumor margins, as captured by a handheld Raman scanner.^[^ ^75^ ^]^ Copyright 2023, Springer Nature. c) Peripheral Raman probe after tumor detection using a handheld Raman scanner.^[^ ^76^ ^]^ Copyright 2023, John Wiley and Sons. d) Imaging contrast diagram.^[^ ^77^ ^]^ Copyright 2019, Theranostics. e) Resection of brain tumors performed in a mouse model.^[^ ^74^ ^]^ Copyright 2019, American Chemical Society.

Desroches et al.^[^ ^73^ ^]^ demonstrated the utilization of a novel puncture needle in a study conducted in 2018, as illustrated in **Figure** **7**a. In addition to obtaining tissue samples in a minimally invasive manner, the needle can also perform real‐time Raman spectroscopy acquisition of the part during puncture. This optical molecular imaging technique can provide the foundation for safe and high‐quality targeted clinical biopsies. Han et al.^[^ ^74^ ^]^ reported a SERRS probe. Figure 7e presents a schematic of the SERRS‐guided glioma surgery, where a SERRS probe containing gold nanocrystals (yellow part in the figure) was injected intravenously, and brain tumor resection was performed in a mouse model using this method. Figure 7b illustrates that the handheld Raman scanning instrument can display tumor images in real‐time. In particular, gliomas were injected into window chambers, e.g., mice (i) and frontal‐ and lateral‐view images of ans‐IR7 (ii). Importantly, this probe distinguished the invasive margin of the tumor xenograft with a high signal‐to‐background ratio. The craniotomy plan was created after preoperative magnetic resonance imaging (MRI) identified the location of the orthotopic glioma xenografts in rat model brains. Using a handheld Raman scanner, the brain tumor was surgically removed piece‐by‐piece until all Raman signals from the probe were lost in the resection bed.

Shams et al.^[^ ^75^ ^]^ developed an in‐situ imaging navigation method for spatially tracked Raman spectroscopic probes. An electromagnetically monitored Raman spectroscopy probe and tracked transrectal ultrasound TRUS imaging with automatically recorded diagnostic MRI comprised the proposed system fabricated by 3D Slicer. McGregor et al.^[^ ^76^ ^]^ developed a miniature Raman probe. As illustrated in Figure 7c, the probe displayed structures around the lung and live Raman spectral information around the normal tissues. This is the first time that Raman spectra of the peripheral lung have been obtained in vivo. The few minutes required to add Raman measurement to a biopsy operation did not create a heavy burden on the patient or medical team. Zhang et al.^[^ ^77^ ^]^ examined the differences between tissue section staining and Raman imaging of laryngeal cancer and normal tissues. Figure 7d (i) shows the Raman image of the laryngeal cancer tissue, and (ii) depicts the hematoxylin‐eosin staining image of the laryngeal cancer tissue. Each unit in the image corresponds to a complete Raman spectrum, which collectively forms a pseudocolor map with the material structure and composition.^[^ ^78^ ^]^

Raman spectroscopy with optical fibers and puncture needle imaging primarily relies on molecular material chemistry for unlabeled, non‐destructive, and real‐time in situ analysis. Different Raman peak intensities can generate images of different configurations, such as molecular structures,^[^ ^79^, ^80^ ^]^ stress images, and crystal images. The first problem to be solved in association with Raman imaging diagnosis is ensuring high consistency with histopathological diagnoses.

**Figure 8:**
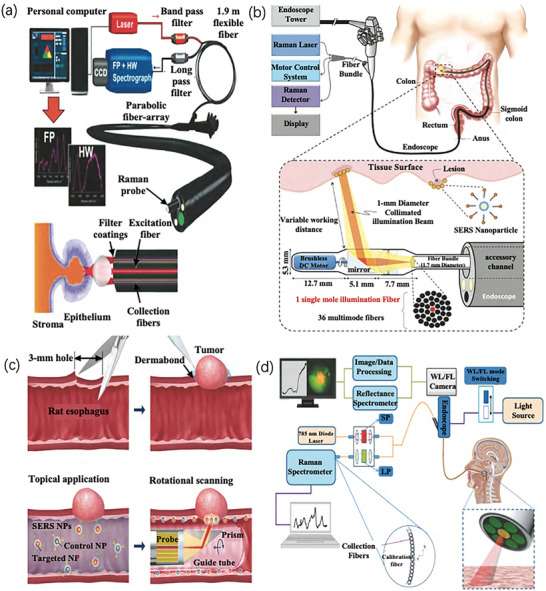
a) Schematic of optical fiber and endoscopic system imaging.^[^ ^81^ ^]^ Copyright 2015, Springer Nature. b) Schematic of Raman spectrum imaging during colonoscopy.^[^ ^82^ ^]^ Copyright 2015, Plos One. c) Schematic of the esophageal tumor model and endoscopic imaging detection in a rat model.^[^ ^83^ ^]^ Copyright 2015, Optical Society of America. d) Schematic of the spectrum and integrated endoscope system obtained by optical imaging Raman spectrum.^[^ ^84^ ^]^ Copyright 2021, Elsevier.

In addition to the application of Raman fiber probes, a combination of optical fibers and endoscopy systems can be used to obtain real‐time imaging information on clinical diseases and perform timely diagnosis. Bergholt et al.^[^ ^81^ ^]^ developed a red‐fiber Raman endoscopy system. **Figure** **8**a depicts the high wavenumber and fingerprint spectra of the colon tissue surface obtained using this system. Garai et al.^[^ ^82^ ^]^ developed a clinical endoscopy system that incorporated a small noncontact optomechanical Raman gadget as a complement. Rapid scans of the circumference of topologically complicated organs with hollow luminal surfaces (such as the colon and esophagus) are made possible by this instrument, which also generates quantitative representations of the current SERS NP relative concentrations. Additionally, by simultaneously detecting the distinctive spectral fingerprints of numerous SERS NPs, this method provides unmatched multiplexing capabilities. The instrument can scan an organ cavity using targeted biomarker SERS particles, as shown in Figure 8b. Similarly, Wang et al.^[^ ^83^ ^]^ revealed that the visualization of the tumor location and quantitative expression of biomarkers were consistent with the data verified by immunohistochemistry and flow cytometry, as depicted in Figure 8c. To target EGFR and HER2, the rat esophageal luminal surfaces were topically treated with antibody‐conjugated SERS NPs. NPs bound to the esophageal lumen were carefully imaged using a small spectral endoscope with rotational scanning and axial pull‐back. Multiplexed SERS NPs can be excited at a single illumination wavelength to guarantee that all NP reporters in the measurement are incorporated uniformly in terms of illumination intensity, area, and depth (785 nm). Second, because these NPs are rather large (120 nm), they can quickly (15 min) create a molecular picture contrast between the tumor and normal esophagus by remaining near the lumen surface, as opposed to dripping into the tissue and becoming stuck. Lin et al.^[^ ^84^ ^]^ combined white‐light imaging with a four‐mode endoscopic system developed using diffuse reflection, spontaneous fluorescence imaging, and Raman spectroscopy, which can be used for in vivo imaging in clinical practice, as illustrated in Figure 8d. Cordero et al.^[^ ^85^ ^]^ introduced a Raman imaging system based on a compact fiber probe to characterize in vitro tumor grading. They created the first in vivo endoscopic cancer detection system that combined white light imaging (WLI), autofluorescence imaging (AFI), diffuse reflectance spectroscopy, and Raman spectroscopy. This study demonstrates that Raman spectroscopy can further complement clinical trials, which is critical for the advancement of in vivo Raman imaging.

The identification of biomarker‐targeting SERS NPs in the human gastrointestinal system can accelerate early cancer diagnosis; however, a clinically practical device with rapid Raman imaging capacity has not yet been demonstrated. Brighter NPs would be beneficial to increase the SNR and, subsequently, the imaging speed. In addition, more efforts are required to further advance spectral endoscopic methods.

**Figure 9:**
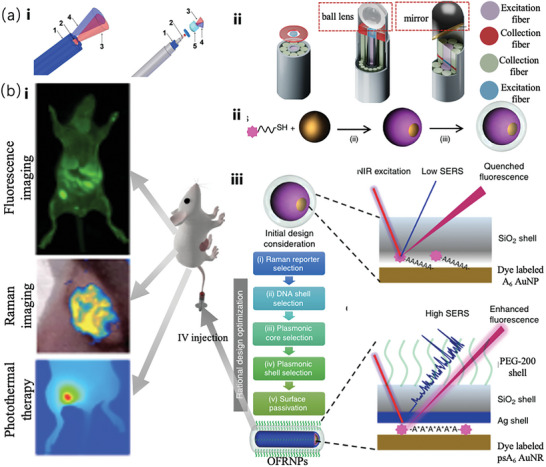
a) Schematic of different types of probes.^[^ ^87^, ^88^ ^]^ Copyright 2020, Analyst, Copyright 2020, Springer Nature and b) FRNP formation and testing using optimized protocols in mouse models.^[^ ^89^ ^]^ Copyright 2019, Springer Nature.

Stomp‐Agenant^[^ ^86^ ^]^ compared superficial and non‐superficial Raman measurements for the diagnosis of bladder cancer and established a Raman histopathology‐based classification model and PCA. **Figure** **9**a presents a schematic of the Raman probe model, where the left and right sides represent the distal tip architectures of the nonsuperficial and superficial Raman probes, respectively (i). On the right, i) shows the fiber collection, ii) the excitation fiber, iii) the cone of the Raman laser, iv) the device used to obtain the Raman signal, and v) the condenser. The endoscopic probes commonly used in clinical practice have distinct characteristics. Cordero et al.^[^ ^87^ ^]^ presented a detailed analysis of endoscopic Raman probes; their primary differences were related to whether they were confocal or volumetric probes. Figure 9a shows the simplest endoscope optical‐fiber Raman probe, that is, the confocal probe (ii). Figure 9a shows that the confocal probe has a significantly larger volume than the optical component of the probe, and the structure is highly complex. Figure 9a shows the capacity to add a prism or ordinary confocal mirror probe. Currently, a common problem with Raman imaging is the point‐by‐point acquisition of spectral information instead of real‐time detection.

Faster imaging is required to cover a clinically important area in real time because existing Raman scanners rely on slow point‐by‐point spectrum acquisition. In recent years, researchers have begun using line scanning as an alternative to traditional point‐by‐point imaging for faster Raman imaging. In point‐by‐point imaging, each pixel must be measured individually, making the measurement slower. Line scanning, in contrast, scans the Raman laser beam along one line of the sample and then acquires data from the other lines by moving the sample or laser beam. This method can significantly accelerate imaging, particularly when the entire region needs to be scanned. By combining line‐scanning techniques, researchers can acquire more image data in a shorter period, leading to faster clinical testing.

Pal et al.^[^ ^88^ ^]^ developed bimodal fluorescent Raman nanoparticles (FRNPs) to solve this problem. Figure 9b (ii) presents a schematic of the particle formation process, which combines the specificity of Raman spectroscopy with the timeliness and versatility of fluorescence imaging. Accordingly, they continuously optimized FRNPs. Figure 9b (iii) shows the traditional and optimized FRNPs, respectively. Pal et al. successfully utilized optimized FRNPs for tumor detection, imaging, resection, and Raman‐based tumor edge cleaning in mouse models, as shown in Figure 9b (i).

To address the issues associated with Raman‐based imaging systems, Placzek et al.^[^ ^89^ ^]^ proposed an imaging system based on piezoelectric‐tube optical coherence tomography and fiber‐optic Raman probes. These probes enable faster imaging speeds because they can acquire multiple data points simultaneously, thereby reducing the required imaging time. The development of these technologies provides strong support for achieving faster clinical Raman imaging, enabling physicians to obtain imaging information in a timely manner for a more accurate diagnosis and treatment. Abramczyk et al.^[^ ^90^ ^]^ used Raman microspectroscopy and endoscopy to detect animal models and surgically remove cancerous tissues. The results demonstrated that the cytochrome C content in cancer cells was lower than that in normal cells. The combination of Raman and fluorescence technologies has significant potential for clinical imaging detection. Paidi et al.^[^ ^91^ ^]^ combined Raman spectroscopy with 3D diffraction tomography to capture the morphology and molecular information of cancer cells at a single‐cell resolution in clinical studies. These results broaden the application of Raman spectroscopy in clinical research.

Currently, different types of Raman technology‐based imaging devices are available on the market. Alix et al.^[^ ^92^ ^]^ investigated the application of a small optical‐fiber Raman system and a standard commercial Raman microscope for the clinical imaging detection of mitochondrial diseases. The results revealed that both are feasible and that the fiber‐optic technique is slightly superior to the Raman microscopy system. Collectively, these investigations demonstrate that optical imaging and spectroscopy are sensitive to variations in cellular states driven by biological activities. The proposed method has several advantages over previous methods that may be used to examine minute molecular variations in isogenic cells, including the capacity to nondestructively probe single live cells and the absence of substantial sample preparation.

Accurate resection of pathological tissues is critical for clinical treatment, and the assessment of intraoperative resection margins remains a major challenge for successful cancer surgery. High precision can be achieved with Raman spectroscopy for the detection of cancer cells; however, this is time‐consuming. Liao et al.^[^ ^93^ ^]^ developed a selective Raman spectroscopy method based on high‐wavenumber fingerprint Raman spectroscopy. Lizio et al.^[^ ^94^ ^]^ developed devices based on Raman and automatic fluorescence spectroscopy for the surgical evaluation of clinical breast cancer. According to Lauwerends, a study published in 2022 demonstrated the advantages of the complementary application of Raman and fluorescence technologies in promoting resection and displaying clear surgical margins in the clinic,^[^ ^95^ ^]^ further verifying the potential clinical application of FRNPs.

Based on Ag NPs and SERS technology, Lin et al.^[^ ^96^ ^]^ performed a surgical evaluation and tumor screening of patients with breast cancer before and after surgery, in addition to healthy volunteers, in a clinical setting. Similarly, Grieve et al.^[^ ^97^ ^]^ analyzed the blood of healthy individuals and patients with cardiovascular diseases using Au NPs and SERS technology. In contrast to the study by Lin et al., Grieve et al. introduced machine learning algorithms to establish systematic disease prediction models in clinical studies more rapidly and conveniently.

**Figure 10:**
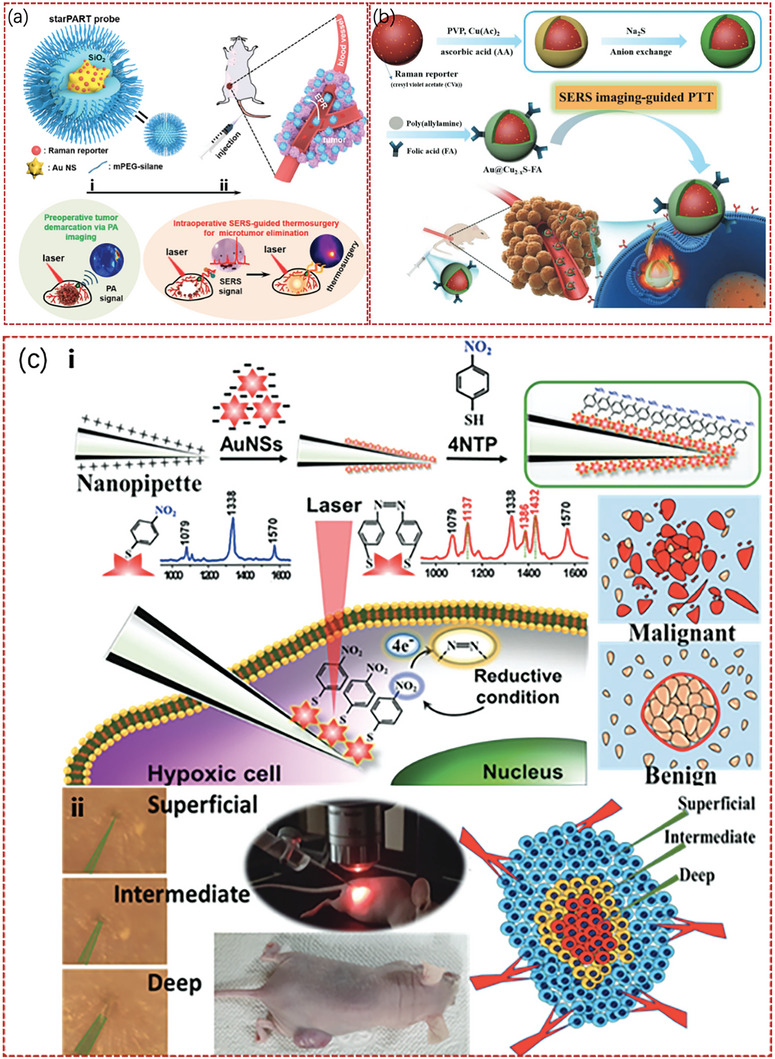
a) Schematic of the structure of the starPART probe and the surgical treatment process using the probe.^[^ ^98^ ^]^ Copyright 2023, American Chemical Society. b) Similar to (a), the schematic of the synthesis of Au@Cu_2_ XS‐FA NPs and the imaging treatment process in the mouse model.^[^ ^99^ ^]^ Copyright 2023, John Wiley and Sons. c) Schematic of the synthesis for the functional nanoprobe and the operating principle of the nanoprobe to detect oxygen levels in cancer cells.^[^ ^100^ ^]^ Copyright 2023, John Wiley and Sons.

Wen et al.^[^ ^98^ ^]^ reported a method involving SERS‐guided thermal surgery to eliminate residual tumors due to disease recurrence caused by incomplete surgical resection. These include photoacoustic (PA), SERS, and thermosurgical (starPART) probes based on Au nanostars. This starPART probe utilizes the important benefits of Au nanostar‐based photoacoustic (PA) imaging, SERS detection, and photothermal tumor ablation. It is composed of an Au nanostar core, a Raman molecule layer, and a silica outer layer. These distinguishing characteristics allow intraoperative SERS‐guided thermosurgery to completely remove any remaining microtumors and preoperative PA imaging for the surgical resection of tumors. **Figure** **10**a illustrates the detailed clinical treatment process using this method. In clinical surgery, this method can completely eradicate microtumors, thus serving as a basis for the development of novel clinical treatments. Specifically, (i) is the StarPART probe that can be targeted for adsorption onto the edge of the tumor using the retention effect and strong permeability, and the outline of the tumor edge can further guide surgical resection, whereas (ii) indicates the continuation of SERS. Furthermore, AL‐237 was used to prepare Au@Cu2xS core‐shell NPs combined with SERS for tumor imaging and resection. These novel Au@Cu2xS NPs offer a promising platform for bimodal imaging‐guided PTT owing to their enhanced physiological stability, good biocompatibility, and PT conversion efficiency. Figure 10b presents a schematic of the synthesis of the substance and its clinical application in a mouse model. This method demonstrates significant potential for diagnosing the aggressiveness of cancer cells.^[^ ^99^ ^]^ Figure 10c presents a schematic of the fabrication of functional nanotubes and a schematic of the Raman probe for detecting the oxygen content of cancer cells, where (ii) presents a comparison of the oxygen levels at different depths detected by nanoprobes in a mouse model.^[^ ^100^ ^]^

The characteristics of SERS with fibers and NPs were incorporated into one unit in the above studies. With deep tissue penetration in SERS detection, concurrent SERS detection modalities have complementary power in pre‐ and intraoperative resections. With improved photoacoustic signals, this new type of SERS‐guided NP is a strong option for precise tumor navigation and nondestructive PT treatment guided in vivo by two optical imaging modes. Additionally, a novel surgical technique based on a “three‐in‐one” theranostic starPART nanoprobe has been reported for image‐guided surgical resection of tumors and the following.^[^ ^101^, ^102^ ^]^

### 3.3 Detection of Markers

#### 3.3.1 Exosomes

**Figure 11:**
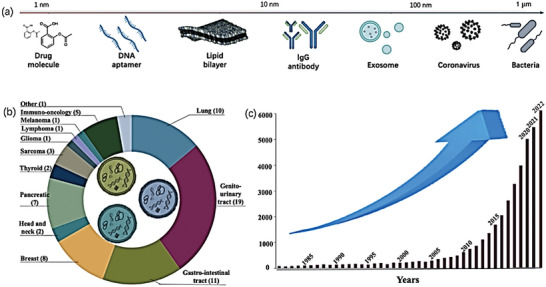
Approximate size range of biological structures and the research status and hotspots of exosomes in diseases. a) Size range of different biological structures.^[^ ^103^ ^]^ Copyright 2023, Springer Nature. b) The proportion of diagnoses of different diseases in exosome‐based clinical trials registered on clinicaltrials.gov. c) Exosomes as markers of clinical diagnosis and treatment have increased from 1980 onward, and their popularity has increased in recent years.^[^ ^104^ ^]^ Copyright 2021, Elsevier.

**Figure 12:**
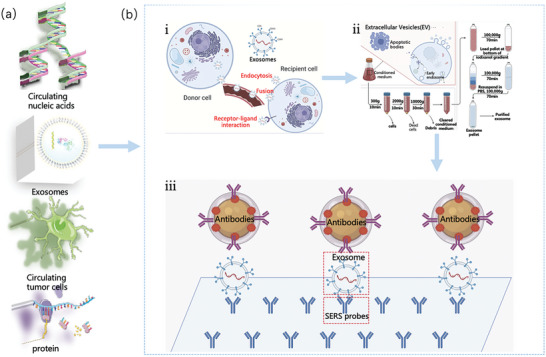
Tumor markers. a) Types of tumor markers include proteins, circulating nucleic acids (NAs), exosomes, and circulating tumor cells (CTCs). b) Exosome formation, extraction, and surface‐enhanced Raman detection.^[^ ^105^ ^]^ Copyright 2023, Elsevier.

The development of readily available and reasonably priced blood‐based biomarkers that can identify the same clinical pathologies in recent years has the potential to alter the diagnosis of clinical diseases worldwide. In clinical diagnosis, diseases are primarily diagnosed by detecting biomarkers in tissue structures or body fluids. The biomarkers of clinical diseases are illustrated in Figure 12a, including proteins, NAs, exosomes, and circulating tumor cells (CTCs), which are classified as disease‐derived substances. Exosomes have been studied extensively. **Figure** **11**a shows the different spatial scales required for tissue structure detection^[^ ^103^ ^]^ and illustrates the status of exosome‐based clinical tests in recent years. Figure 11c presents an increasing trend in studies related to exosomes from 1980 onward, and Figure 11b presents the diagnostic status and proportion of exosomes in different diseases in clinical practice.^[^ ^104^ ^]^ Moreover, the miRNAs identified in this study can be used as biomarkers for clinical diagnosis and Raman technology‐based treatments.

As shown in **Table** **2**, Raman spectroscopy has been increasingly used in clinical applications in recent years to facilitate early diagnosis, clinical treatment, and prognosis. **Figure** **12**b ^[^ ^105^ ^]^ depicts the exosome formation, extraction, and surface‐enhanced Raman detection process: (i) outside the body during the process of organism formation, (ii) the secreted body outside the extraction process, and (iii) the use of Ag NP‐enhanced Raman signals. Exosomes are, in actuality, extracellular vesicles (EV).^[^ ^106^ ^]^ Exocrine secretions were excluded, and vesicles included micro‐vesicles and apoptotic bodies.^[^ ^107^ ^]^ Initially, several researchers reported that exosomes are only flat or cup‐shaped wastes generated after the maturation of red blood cells and are distributed in human body fluids, such as tears, blood, urine, saliva, cerebrospinal fluid, and thoracic spinal fluid.^[^ ^108^, ^109^ ^]^ However, exosomes from different sources contain different biomacromolecules, such as proteins and lipids, which are involved in cell information transmission and function.^[^ ^110^ ^]^ Therefore, they play a critical role in the incidence, development, invasion, and metastasis of diseases and can be used as markers in disease diagnosis and as targets for cancer treatment. Biomarkers for various diseases in clinical practice primarily include substances from diseases.

**Table 2:** Review of the application of Raman spectroscopy to typical clinical diseases over the past 3 years, in terms of the sample type, detection method, and detection results.^[^ ^126^, ^127^, ^128^, ^129^, ^130^, ^131^, ^132^, ^133^, ^134^, ^135^, ^136^, ^137^, ^138^, ^139^, ^140^, ^141^, ^142^, ^143^, ^144^, ^145^, ^146^, ^147^, ^148^, ^149^, ^150^, ^151^, ^152^, ^153^, ^154^, ^155^, ^156^, ^157^, ^158^, ^159^, ^160^, ^161^, ^162^, ^163^, ^164^, ^165^, ^166^, ^167^, ^168^, ^169^, ^170^, ^171^, ^172^, ^173^, ^174^, ^175^, ^176^, ^177^, ^178^, ^179^, ^180^, ^181^, ^182^, ^183^, ^184^, ^185^, ^186^, ^187^, ^188^, ^189^, ^190^, ^191^, ^192^, ^193^, ^194^ ^]^

Clinical Disease Diagnosis	Sample Type	Detection Method	Detection Results	Reference
Ovarian cancer	Patients with plasma	An ensemble model of ternary classification based on machine learning was established and Raman test was successfully performed.	The method can be used for cancer identification.	[126]
Lung cancer	Exosomes secreted by lung normal, cancer cells, and patients’ plasma	The gold nanoparticle (GNP) colloid solution was covered with 3 aminopropyl triethoxysilane (APTES), and SERS was combined with principal component analysis (PCA)‐linear discriminant analysis and partial least squares discriminant analysis (PLS‐DA).	Stage I lung cancer patients were detected using a deep learning model supervised by exosomes.	[127]
	Exosomes secreted by lung normal and cancer cells	Au NPs were prepared to form nano gaps and detected by PCA analysis.	Contributed to studies on exosome surface protein markers for cancer diagnosis.	[128]
	Exosomes secreted by lung cancer patients’ serum (4 µL)	Anti‐PD‐L1 antibody‐modified Au@Ag@MBA SERS tags.	The quantified exosome PD‐L1 biomarker was isolated from serum, and as demonstrated, it can induce the release of multiple signals, thus achieving ultra‐high sensitivity detection.	[129]
	Lung squamous cell carcinoma (LSCC) tissue and healthy lung tissue	Raman spectroscopy combined with multivariate analysis for the microwave ablation of lung cancer.	SERS, combined with the PCA‐linear discriminant analysis (LDA) and leave‐one‐out cross‐validation (LOOCV) methods, was used to accurately distinguish lung cancer tissue from healthy tissues and study the related biochemical mechanism.	[130]
	Serum samples from different types of lung cancer	Ag NPs were used as the substrate to enhance the Raman signal.	The Ag NP SERS technology combined with PCA and PLS‐DA is a promising tool for detecting and screening biomolecular diversity in cancer detection.	[131]
	A clinical blood sample from a lung cancer patient	Gold nanoclusters of sea urchins were synthesized by Ag seed‐mediated growth.	This method can be used to detect mutated genes in lung cancer with high efficiency and specificity.	[132]
	Serum samples from lung cancer patients and healthy people	Combination of Raman spectroscopy and data ablation.	Fourier transform infrared spectroscopy combined with Raman spectroscopy based on data fusion and wavelet transform can effectively diagnose lung cancer patients.	[133]
Breast cancer	Exosomes secreted by HEK‐293T cell MCF‐7 cell and patients’ plasma	The TP‐Au NP probe was developed based on the attraction principle between a negatively charged deoxyribonucleic acid (DNA) tetrahedron and positively charged Au NPs.	Distinguished exosomes were extracted from the plasma of healthy individuals and breast cancer patients.	[134]
	Exosomes derived from breast cancer cells	A plasmonic gold nanopillar SERS substrate (3 × 3 mm) by maskless reactive ion etching (RIE).	Quantitative detection of exosome miRNA was used to detect cancer diseases.	[135]
	Plasma samples from patients with breast cancer at different stages	Raman spectroscopy was combined with multivariate data analysis to analyze the spectra of breast cancer and healthy volunteers.	It was confirmed that the PCA scatter plots of breast cancer patients were different from those of healthy volunteers and breast cancer patients at different stages.	[136]
	Residual microscopic tumor of breast cancer	A three‐in‐one nanoprobe was designed for the image guidance of surgical resection and thermal surgical elimination.	The advantages of PA imaging SERS detection and photothermal tumor ablation significantly improved the surgical results.	[137]
	Breast cancer tissue	The biochemical components of microcalcification foci in breast cancer were studied by Raman spectroscopy and multivariate analysis.	Calcified hydroxyapatite (HAP) was successfully obtained from tumor tissue, with numerous biological macromolecules.	[138]
Pancreatic cancer	Exosomes secreted by pancreatic normal and cancer cell	SERS, label‐free SERS detection, principal component, and differential function analyses.	Exhibited up to 87% and 90% predictive accuracy for healthy control and individual pancreatic cancer samples.	[139]
	Exosomes secreted by pancreatic ductal adenocarcinoma, chronic pancreatitis, normal control plasma	Fe_3_O_4_ @Ag‐DNA‐Au@Ag@DTNB (SERS tag) conjugates label‐free SERS detection.	MicroRNA‐10b in the blood samples can distinguish pancreatic cancer from chronic pancreatitis and normal controls.	[140]
	Exosomes secreted by pancreatic cancer patient serum (4 µL)	Locked nucleic acid (LNA)‐modified Au@DTNB, Fe3O4@TiO2 NP.	Exosome miRNA‐10 can be used to accurately distinguish ductal pancreatic cancer patients from healthy volunteers.	[141]
Melanoma	Melanophilia occurs naturally in the feathers of 26 species of birds	The quantitative analysis of benzothiazole (BT) and benzothiazole (BZ) was performed by Raman spectroscopy combined with stoichiometry (MCR‐ALS).	Raman spectroscopy combined with multivariate analysis can directly identify and quantify melanotropic sub‐units in biological samples.	[142]
	MM DN and composite Nevus (CN)	Raman spectroscopy (RS) and multivariate analysis (MA)	Raman spectroscopy and MCR‐ALS‐based methods were first proposed and used to locate and quantify melanin and DHICA.	[143]
Alzheimer's disease (AD)	Retinal specimen of human	The Raman technique was used to compare imaging of amyloid precursor proteins and mutants in mice or humans	Demonstrated that subtle biochemical changes that occur in the retina may lead to the recognition of Alzheimer's disease (AD).	[144]
	Amyloid β (1‐42) peptide (Aβ42)	Raman technology was combined with a hollow core photonic crystal fiber (HCPCF) to measure FERS, and then FERS was combined with SERS to further amplify Raman signal.	The HCPCF‐based platforms can provide strong Raman signals, thus serving as a basis for the early labeling sensitive diagnosis of AD.	[145]
	Phosphorylated tyrosine, serine, threonine, or histidine residues	An in situ method for protein phosphorylation fingerprinting was developed without antibodies and labels.	The method was ultra‐sensitive in identifying S396 phosphorylated at unit points in Tau410, which is a critical biomarker of AD.	[146]
	Serum of a patient with AD	SERS combined with multivariate statistical analysis. Colloidal silver NPs were used as SERS active substrates.	The method can be used for non‐invasive detection and screening for AD in a clinical laboratory setting.	[147]
	Cerebrospinal fluid samples	A method based on cerebrospinal fluid near‐infrared Raman technology and machine learning was proposed for the diagnosis of AD.	The method proposed in this paper has significant applicability to the clinical diagnosis of AD and can supplement the existing clinical detection methods.	[148]
	Tau protein	A magnetic polystyrene gold (or silver) nano‐composite particle coated with streptavidin was developed, which can be used as a substrate for SERS.	This method has significant potential for the highly sensitive and selective analysis of markers in complex biological samples.	[149]
	Serum obtained from rats fed either a standard diet or a high‐fat diet	PLS‐DA was combined with recipient characteristic curve analysis.	The Raman technique can serve as an effective method for the detection and identification of AD.	[150]
Liver cancer (Hepatocellular carcinoma (HCC))	Purification of serum protein	The serum protein purification method and SERS method based on Ag NPs were combined to screen liver tumors.	The method has significant applicability for the detection and screening of liver cancer.	[151]
	Liver cells	Raman spectral imaging and Matrix‐assisted Laser desorption ionization imaging mass spectrometry (MALDI IMS).	Raman, MALDI IMS, and their combinations exhibit a high potential in addressing specific problems in the diagnosis of liver cancer.	[152]
	Hepatocellular carcinoma and healthy liver tissue	Raman response of mtVDA‐coated untargeted and targeted cetuximab polymer nanocomplexes in mouse hepatocarcinoma tumor tissue.	Raman spectroscopy was used to stratify anticancer nanomedicine to monitor and treat hepatocellular carcinoma (HCC).	[153]
	Liver cells	Over 2000 valid spectra were identified according to the Preferred Reporting Program for Systematic Evaluation and Meta‐analysis (PRISMA) guidelines, and the performance of the analyzed spectra was tested using a random effects model.	This paper further demonstrated the accuracy, specificity, and sensitivity of RS in the diagnosis of HCC.	[154]
Coronary heart disease	Urine	This paper proposes the use of SERS to identify the composition and content of human urine, to diagnose coronary heart disease.	Human urine measurement based on SERS was proposed for the diagnosis of coronary heart disease.	[155]
Bladder cancer	Paracystium	The heterogeneity of high‐fluorescence bladder tissues was assessed, and multivariate statistical analysis was performed on high‐fluorescence and low‐fluorescence bladder tissues, in addition to non‐tumor tissues.	As demonstrated, the strength of lipid collagen and protein strips can be used to track biopsied specimens.	[156]
	Metabolites present in urine	SERS was used to detect urine and predict the tumor grade of bladder cancer.	It was confirmed that SERS is highly applicable to the detection of urine supernatants and precipitates, for the diagnosis of bladder cancer.	[157]
	Paracystium	We compared Raman measurements from a superficial probe with those of a non‐superficial probe, for the diagnosis of bladder cancer.	Raman spectroscopy provides additional information to histopathology.	[158]
	Bladder biopsy	An imaging system based on an optical coherence tomography (OCT)‐probe and optical fiber RS‐probe was proposed to detect and grade bladder cancer and is suitable for the large‐field imaging of bladder cancer.	It indicates that this method has the potential for application in vivo in the future. Combined with an OCT and RS fiber probe, it can effectively diagnose bladder cancer.	[159]
	Cancer bladder specimen	By combining fluorescence imaging with a white light cystoscope, 3D imaging was performed to determine the target region for Raman diagnosis.	Clinical Raman imaging can be used to reveal cancer margins and aid in cancer excision.	[160]
Colorectal cancer (CC)	CC cells vs artesunate (ART)‐treated cells	The combination of RS with biological assay methods can be used to distinguish CC cells from ART‐treated cells.	Raman spectroscopy has been increasingly applied in the biomedical field in combination with biological mechanisms.	[161]
	CC cells	Rapid diagnosis by Raman spectroscopy.	The role of the Raman technique in endoscopy and liquid biopsy was demonstrated, and prospective applications were investigated.	[162]
	Biomedical samples	The use of high‐performance computers to demonstrate the advantages of Raman technology for colorectal cancer detection was demonstrated.	The proposed method is proposed for disease detection; however, it can be used for other applications and spectral‐type data sets.	[163]
	Normal CCD18‐Co, CRL‐1831 and cancer CaCo‐2 human colon cell lines	The label‐free Raman technique demonstrates a high confidence level in normal and human colon cancer cells.	This Raman technique is highly applicable to clinical cancer diagnosis.	[164]
	Protein substrates	Cr^3+^ ion aggregation Au@Ag NPs (ICNPs) was used to establish the CRC protein marker NDKA, and SERS technology was used for detection.	This method provides the advantages of high sensitivity and reproducibility, with high clinical applicability.	[165]
	Live single cells from colorectal adenocarcinoma cell	The Raman technique was used to study the uptake and degradation of a novel photosensitizer DC473 in colorectal adenocarcinoma cells.	This study demonstrated the potential of Raman spectroscopy to investigate the pharmacodynamics of time‐dependent single cells.	[166]
	Serum samples obtained from patients undergoing colonoscopies	We used Raman spectra recorded using 184 serum samples obtained from patients undergoing colonoscopies.	A high‐precision CRC prediction model with a high R‐value was constructed, and its accuracy was verified using a large amount of data.	[167]
	The serum from patients with colorectal cancer (CRC).	In this study, a simple, high‐speed, and low‐cost optical sensing platform was developed to detect the mixture of gold NPs and serum of colorectal cancer patients using SERS technology.	Support vector machine can be used to compute the value of carcinoembryonic antigen and the value of SERS spectrum editing, such that improved prediction results can be obtained.	[168]
	miRNA	A nano‐biosensor with SERS capability is introduced.	These sensors are suitable for emerging just‐in‐time applications.	[169]
Prostate cancer (PCa)	The urinary exosome miRNAs	In this paper, SERS technology based on 3D nanostructures was proposed to detect miRNA.	The SERS technology based on 3D nanostructures is more convenient, accurate, and rapid at detecting miRNA, and it can diagnose numerous types of diseases.	[170]
	Extracellular vesicles (EVs)	In this paper, the extracellular sacs were from the plasma of prostate patients and controlled patients by differential separation.	The Raman effect mediated by Ag NPs significantly enhanced the Raman signal and provided the optimal separation of Raman and SERS spectra for cancer and control group patients.	[171]
	Docetaxel‐based chemotherapy	In this paper, SERS was used to establish a model to predict the response of mCRPC patients to docetaxel chemotherapy.	The SERS technique of serum plays a critical role in clinical applications.	[172]
	A serum specimen from a patient with prostate	Preoperative serum samples of patients with biopsy GG1 and subsequent radical prostatectomy were analyzed using SERS.	It was confirmed that the combination of SERS and PCA‐LDA can be considered adjuvant therapy in clinical practice.	[173]
	Urine	In this study, Raman spectroscopy and a rolling machine neural network were combined for the non‐invasive detection of PCa.	This technique exhibits potential as a method for the early diagnosis of PCa and promotes the application of artificial intelligence in clinical medicine.	[174]
	Lymphocytes and genomes	This paper reports a novel method based on Raman technique. Blood samples from 42 PCa patients were collected and tested using the Raman technique after in vitro irradiation.	Raman spectroscopy was confirmed to distinguish between the toxicity of normal tissues after radiation.	[175]
	Malignant tissue in‐situ	A 3D slicing machine based on an electromagnetic tracking Raman spectrum probe was realized, combined with transrectal ultrasound (TRUS) imaging and magnetic resonance imaging (MRI).	As demonstrated, Raman spectroscopy can be used to determine the prosthesis and human prostate tissue in situ, to confirm the location of prostate lesions in clinical settings.	[176]
	Cancer cells.	Comparison of Raman spectra of PCa cells pre‐irradiation (physicochemical damage model) and post‐irradiation (biological response model) with respect to their biochemical compositions.	The Raman technique was confirmed to distinguish initial cell damage from cell response and support cell pre‐treatment.	[177]
	Lipid droplets (LDs)	The analysis involved a comparison of the LDS composition of the original PCa cells with those subjected to X‐ray irradiation.	The significant influence of LDs in the process of cell response was confirmed, and the time dependence of this phenomenon was determined.	[178]
	NPs‐serum mixture	This paper proposes a novel serum strategy, namely, SERS technology based on the coffee ring effect to screen different types of cancer.	As demonstrated, SERS combined with partial least squares/support vector machine (PLS‐SVM) and the coffee ring effect demonstrates significant potential for cancer detection.	[179]
	Dietary fatty acid (FA)	The combination of Raman microscopy and commercial shear‐flow microfluidic systems resulted in a novel experimental system for the monitoring of fatty acid uptake in PCa (PC‐3).	Based on the experimental system proposed in this paper, docosahexaenoic acid and eicosapentaenoic acid were found to inhibit palmitic acid and arachidonic acid uptake by PC‐3 cells.	[180]
	PC‐3 PCa cells	The dose and time‐dependent response of PC‐3 PCa cells to X‐ray exposure was investigated under clinical conditions.	Raman spectroscopy was verified as an effective auxiliary tool for clinical ionizing radiation therapy.	[181]
	Patient cohort	In this paper, a large number of patients were tested at different latitudes.	The fitting of the Raman fitting peak and individual strength characteristics can enhance the classification performance.	[182]
	Prostate‐specific antigen (PSA)	The differences in Raman spectra between serum samples with normal and changed PSA values were examined, and PCA and PLS were combined to analyze the differences.	As demonstrated, Raman spectroscopy can be conducted to screen PSA patients and detect follow‐up treatment.	[183]
	Blood stream	Surface‐enhanced Raman scattering technology without labels was used to collect the serum of prostate patients for diagnosis, and a CNN based on Letnet‐5 was then constructed to identify PCa patients.	As demonstrated, the combination of convolutional neural network classification (CNNC) and serum for SERS analysis allowed for the identification of prostate patients. The development of larger data sets in future research will complement PCa bone scanning techniques.	[184]
	Cancer biomarkers in serum samples	Diethylenetriamine was used as a crosslinking agent and tannic acid was used as a functional monomer, as a template molecule of prostate antigen.	This method was confirmed to detect prostate patients specifically and sensitively in clinical applications.	[185]
	PCa cells	A PLSR model was developed to demonstrate the cytochemical changes after radiation exposure. Raman mapping of the entire cell region allows for a more complete understanding of the changes caused by X‐rays.	The advantages of a mapping technique over a single point measurement were verified by comparing the corresponding PLSR models.	[186]
Lyme disease	Urine	A urine‐based screening technique was developed for Lyme disease (LD).	This study was verified as statistically significant for the urine detection of LD‐positive patients.	[187]
Celiac disease (CD)	Tissue samples	Diagnosis requires a combination of specific serology and typical duodenal lesions.	A non‐invasive method was demonstrated for the diagnosis of celiac disease by analyzing serum samples using the Raman technique.	[188]
Gastric cancer	This adenocarcinoma of the gastric mucosa is normal	The optical fiber Raman spectroscopy system was combined with the spectral integral energy ratio matched by a gastroscope	A method wasp proposed for rapid diagnosis of gastric cancer.	[189]
	Gastric cancer samples	It was further confirmed that the combination of Raman‐based endoscope and fiber optics has significant potential for the in situ real‐time diagnosis of tumors.	It was confirmed that the Raman technique demonstrates a significantly broad application prospect for the diagnosis of gastric cancer.	[190]
	Urine	The influence of differently sized Au NPs on urine was evaluated.	The results exhibited that SERS technology based on gold NPs exhibits significant potential for the detection of gastric cancer and breast cancer.	[191]
	Blood serum	The Raman technique was used to distinguish between serum samples from healthy volunteers and patients with gastric cancer.	It was preliminarily confirmed that the detection of serum by Raman spectroscopy is a promising method for the diagnosis of gastric cancer	[192]
Hirschsprung disease	Detection of ENS in human intestine	Raman spectra of various layers of the gastrointestinal wall of human rectal specimens were collected.	Endoscopic Raman spectroscopy can serve as a novel non‐invasive diagnostic tool for human vascular endothelial cells.	[193]
MIA PaCa‐2 Pancreatic cancer tumor	Tumor regenerative cells (TRCs) and parental control cells	Machine learning was used to analyze the Raman spectra of the MIA PacA‐2 human pancreatic cancer cell lines to distinguish parental control cells from tumor regenerative cells (TRCS), and to reveal molecular characteristics.	It was confirmed that the combination of the Raman technique, machine learning, and statistical analysis exhibits a high applicability to pancreatic cancer research.	[194]

#### 3.3.2 Nucleic Acid (NA)

Numerous virus‐associated disorders, including those caused by the most recent pandemic due to COVID‐19, have been detected using NA biomarkers.^[^ ^111^ ^]^ The phenomenon of CRISPR‐Cas‐based transactivation has demonstrated excellent potential for the development of sensitive and selective NA detection. An ultrasensitive detection method using SERS was used to create a biosensor without NA amplification based on CRISPR‐Cas12a. They combined a CRISPR‐Cas12a system activated by viral DNA with a Raman‐sensitive system composed of ssDNA‐immobilized Raman probe‐functionalized Au nanoparticles (RAuNPs) on a graphene oxide (GO)/triangle Au nanoflower array. This CRISPR‐based Raman‐sensitive technique enhances the detection sensitivity for multiple viral DNAs. Recently, SERS has become a popular method for biosensing in cancer theranostics. In virtually all instances, conventional colloidal dispersions of silver and gold NPs or nanostructured films have been used as plasmonic substrates for direct SERS measurement of NAs.^[^ ^112^ ^]^ SERS has considerable potential for clinical cancer detection and subtyping when combined with genetic probes to enable the ultrasensitive and multiplex detection of tumor‐derived NAs.

In our opinion, future efforts should focus on creating novel classes of strong and highly effective plasmonic substrates that meet the demands of NA analysis in solution. Ideally, these materials should be produced using straightforward, standardized processes and should exhibit high levels of experimental adaptability and robust responses when exposed to a variety of environments, with little to no changes in their plasmonic and colloidal properties. As with other large biomolecules, SERS applications for NAs often use indirect methods to identify and quantify target strands.

#### 3.3.3 Protein

Proteins are the material basis of life and the carrier of life activities, and many clinical diseases are accompanied by changes in protein levels. There are two main approaches for determining proteins: indirect and direct. An ideal SERS sensor must contain antibodies or other target molecules to create a stable and cohesive system for indirect techniques. Sensors must be able to process data repeatedly so that the results can be compared after the analysis. Therefore, a variety of sensing arrangements have been devised and used in the SERS‐based quantitative analysis of proteins to produce a highly sensitive detection method.^[^ ^113^ ^]^ A Raman system made up of a substrate and a Raman spectrometer simplified the identification and measurement of biomarker proteins. As a result, the type of label selected as well as the marking technique should be tailored for each application. Partial clinical diseases are characterized by the intracellular or extracellular aggregation of misfolded proteins.

Different proteins can be used as markers for the detection of corresponding diseases, such as amyloid‐β^[^ ^114^ ^]^ and tau in Alzheimer's disease (AD),^[^ ^115^ ^]^ α‐synuclein^[^ ^116^ ^]^ in Parkinson's disease, and TAR DNA‐binding protein^[^ ^117^ ^]^ in amyotrophic lateral sclerosis. Before dementia starts in AD, amyloid‐ β (Aβ) begins to collect and accumulate in cortical extracellular plaque. Tau aggregates propagate from the medial temporal lobe and accumulate heavily in the neocortex of AD patients in a stereotypical manner. Aβ’s pathology most likely controls or at least facilitates this process. All autosomal dominant types of AD are brought on by mutations in the genes encoding proteins associated with Aβ turnover; these proteins encourage Aβ aggregation, which leads to extensive tau‐tangle disease and cognitive impairment. Data from individuals with mutations in the presenilin 1 (PSEN1), presenilin 2 (PSEN2), and amyloid precursor protein (APP) genes from families involved in the dominantly inherited Alzheimer's network were analyzed.

Moreover, graphene‐based materials (GBMs) have found significant traction in biomedical applications spanning diverse domains, such as antibacterial agents, photothermal anticancer therapies, tissue engineering, targeted genes, drug delivery, bioimaging, and biosensing. These applications have generated considerable interest in the nanomedicine landscape. The integration of graphene analysis with Raman spectroscopy has led to substantial advancements in this field. Double‐sided graphene not only enhances Raman scattering and fluorescence quenching in biostructures but also couples these two signals, thereby improving the sensitivity and accuracy of detection.^[^ ^118^ ^]^ Graphene‐assisted Raman spectroscopy has been applied to rapid biomarker screening for AD, where accurate detection and identification of specific biomarkers have been achieved by enhancing the Raman signals in samples.^[^ ^119^ ^]^ Graphene has also been used to study the charge transfer process between amyloid β and graphene and the fibrillation process of amyloid β.^[^ ^120^, ^121^ ^]^ This study demonstrates the key role of graphene in probing protein interactions and molecular structural changes, providing detailed information for a deeper understanding of biomolecular properties.

#### 3.3.4 CTCs

CTCs are shed from the tumor tissue into blood vessels and travel via the bloodstream to infiltrate other organs, causing lethal metastases. Identification of CTCs is of enormous scientific significance for early diagnosis, quick assessment of therapeutic efficacy, in vivo drug resistance testing, customized treatment, tumor recurrence detection, and survival time judgment, among other therapeutic applications. CTC detection is an FDA‐approved liquid biopsy technique, owing to its comparatively high specificity. Cell culture, phenotypic analysis, and genotyping can all be used to investigate CTCs in vitro. Since there are only 1–100 cancer cells per milliliter of blood at metastases, it is difficult to isolate and detect these cells, necessitating the use of ultrasensitive techniques.^[^ ^122^ ^]^

Methods of CTC isolation include^[^ ^123^ ^]^:

1. Density‐based cell separation size
2. Negative selection of leukocytes by antibodies directed against hematopoietic cells or by erythrocyte and leukocyte depletion.
3. Magnetic separation using magnetic beads with antibodies against tumor‐specific markers added to them.
4. Separation based on migratory characteristics, size, charge, and deformability.

Despite the well‐established accuracy of immunofluorescence, this method has some well‐known flaws, including quenching of fluorescence signals upon excitation and numerous false positives caused by non‐specific antibody absorption,^[^ ^124^ ^]^ There is yet no “gold standard approach” for CTC detection that satisfies high specificity, sensitivity, and accuracy. CTCs can be found in blood samples using SERS techniques on a microfluidic chip. Compared to the fluorescence method, SERS provides a strong, narrow, and sharp fingerprint‐like signal that is easily recognizable even under extremely complex biological conditions.

A tissue sample is obtained from a clinical setting using a needle, endoscope, or an open surgical procedure. The tumor sample is then molecularly analyzed to help doctors determine the diagnosis, prognosis, and course of treatment. However, tissue sampling is intrusive, dangerous, expensive, uncomfortable, and not always feasible.^[^ ^125^ ^]^ In recent years, the characterization of biomarkers analysis in a peripheral blood sample, which serves as a “liquid biopsy,” has gained special attention. A popular area of research is the advancement of methods for precise biomarker identification. SERS tags are appealing candidates for a variety of applications, including in vivo detection and point‐of‐care (POC) devices, owing to the ultrasensitivity of SERS.

### 3.4 Latest Clinical Follow‐Up

Raman spectroscopy can discern the heterogeneity among tumor cells through chemical composition analysis, subcellular structure examination, cellular metabolism assessment, and cellular response evaluation to drugs. It enables the acquisition of information pertaining to the protein and NA content within the tumor tissue. During the early stages of cancer, relatively diminished levels of proteins and NAs are typically observed, which may increase as the cancer progresses. These alterations reflected the dynamic processes of cell proliferation and metabolic activity. Additionally, various stages of cancer are typically accompanied by shifts in lipid composition. Raman spectroscopy is instrumental for identifying lipid types and quantifying their content in tumor tissues. This information contributes to our understanding of the membrane structure and distinctive metabolic activities of cancer cells. Raman spectroscopy facilitates the detection of metabolites and their concentration fluctuations, including those closely linked to cancer metabolic activity, such as lactate and glutamate. Changes in the concentrations of these metabolites can be used to distinguish between different cancer stages and potentially serve as biochemical markers for clinical staging. As cancer progresses, the tumor tissue often requires an increased vascular supply to support its growth. Raman spectroscopy can be employed to identify angiogenesis markers such as hemoglobin, aiding in the determination of tumor progression stages. In summary, Raman spectroscopy is a non‐invasive approach for enhancing our understanding of cancer staging and progression by analyzing the biochemical components in tissue samples or biological fluids. These hold promise for clinical applications, including the development of personalized treatment strategies and prognostic prediction. Relevant studies pertaining to various types of cancer, listed in Table 2, provide readers with a more comprehensive understanding of the dynamic research landscape in this field.

As shown in the table above, a variety of clinical diseases have been the subject of Raman spectroscopy studies, with cancer being the most prominent. These studies share the following characteristics: the need for better early detection, high sensitivity and specificity, a well‐characterized disease process, and relatively easy access to the organs under study. In the following sections, we elaborate on the use of Raman spectroscopy for breast cancer and PCa.

#### 3.4.1 Breast Cancer

**Figure 13:**
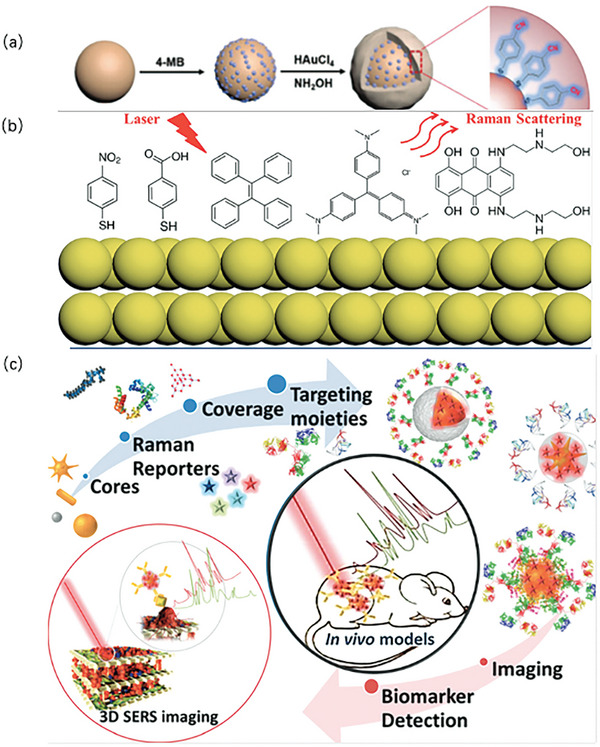
a) Schematic of the formation of surface‐enhanced NPs with an intermediate layer of 4‐MB.^[^ ^199^ ^]^ Copyright 2023, Journal of Materials Chemistry B. b) Formed NPs bound to aromatic amino acids. c) Action process of this NP mixture in a mouse model.^[^ ^200^, ^201^ ^]^ Copyright 2019, American Chemical Society, Copyright 2016, Elsevier.

Microcalcification is a primary indicator of cancerous lesions. Marro et al.^[^ ^195^ ^]^ used Raman spectroscopy to study breast cancer tissue sections and analyze the biochemical components of microcalcifications in breast cancer. DNA is wrapped in hydroxyapatite (HAp) microcalcification substances, and several other biological macromolecules are involved in the calcification of proteins, lipids, and polysaccharides. This led to research on the role of Hap in malignant tissues. Kopec et al.^[^ ^196^ ^]^ employed Raman spectroscopy and Raman imaging to distinguish between tumor markers. The bands of carotenoids (1156 cm^−1^, 1520 cm^−1^), proteins (1004 cm^−1^), fatty acids (1444 cm^−1^, 1655 cm^−1^), and cytochrome c (1585 cm^−1^) can be used as universal biomarkers for the assessment of human tumor aggressiveness. This study demonstrated the potential of Raman spectroscopy for clinical applications in tumor diagnostics. Aromatic compounds exhibit a wider Raman scattering range than aliphatic chains; thus, aromatic compounds can be used as markers in Raman spectra. **Figure** **13**a shows that 4‐MB was placed between the two Au NP cores and shells, which enhanced the Raman signal. Contorno et al.^[^ ^197^ ^]^ discussed the feasibility of using aromatic amino acids as markers for breast cancer diagnosis using Raman spectroscopy. Moreover, they found that aromatic amino acids were overexpressed in breast cancer cells and that their content was significantly different from that in healthy tissues. Figure 13b presents a schematic of the SERS dye formation, and Figure 13c presents the process of marker detection in the mouse model.

Low serum albumin levels are associated with breast cancer. Therefore, serum albumin levels can be used as a prognostic marker for breast cancer. However, traditional detection methods such as the surface plasmon resonance method for serum albumin detection, which is a fluorescence method, involve complicated operational steps, low efficiency, and poor stability.^[^ ^198^, ^199^ ^]^ The traditional separation methods for serum albumin exhibit toxic effects, require expensive instruments, and result in poor reproducibility.^[^ ^200^ ^]^

**Figure 14:**
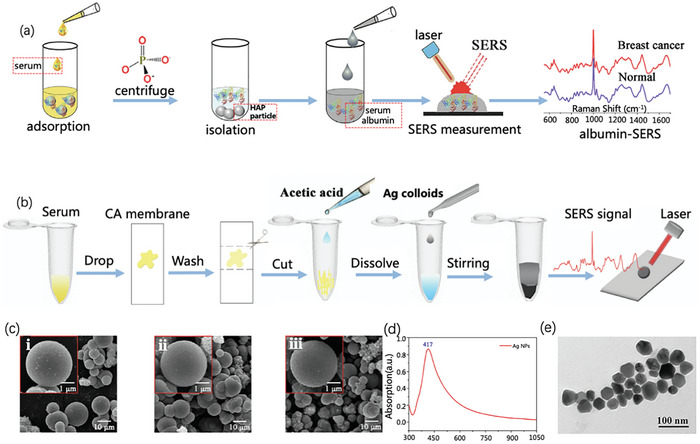
Diagnosis of breast cancer using serum albumin detection. a) Purification and extraction of serum albumin by adsorption of HAP.^[^ ^201^ ^]^ Copyright 2023, John Wiley and Sons. b) Extraction and purification of serum albumin using a cellulose acetate membrane (CA). c) Effect of HPA on serum albumin under a scanning electron microscope. d) Raman signal is strongest when Ag NPs are 417 nm. e) The TEM imaging of Ag NPs.^[^ ^202^ ^]^ Copyright 2023, Elsevier.

Lin^[^ ^201^ ^]^ proposed a novel method for extracting serum albumin, as shown in **Figure** **14**a, in a report published in November 2021. Using this method, albumin was extracted and purified by the adsorption and release of serum albumin by HAP and diagnosed using SERS to detect breast cancer. Figure 14c shows the SEM image of HAP in the process of adsorption and release of serum albumin: (i) the original state, (ii) the state of adsorption of serum albumin, and (iii) the state of release of serum albumin. In a subsequent publication, Lin et al.^[^ ^202^ ^]^ proposed a method for extracting and purifying serum albumin using a fiber acetate membrane (CA) (Figure 14b). Accordingly, serum albumin was first adsorbed onto the CA, and the CA membrane was then cut into small pieces to extract serum albumin. Ag NPs of different sizes were used to obtain the maximum Raman signal during the SERS step. As shown in Figure 14d, the Raman signal was the strongest at 417 nm using this method. Simultaneously, PCA‐LDA and PLS‐SVM were combined, and the results revealed that the latter was more suitable for the diagnosis of breast cancer. Both methods use Ag NPs to enhance the Raman signals. Figure 14e presents a transmission electron microscopy (TEM) image of Ag NPs.

Lin et al. further demonstrated the potential of unlabeled SERS technology combined with the purification and detection of serum proteins using an acetate membrane for the diagnosis of breast cancer.

This section demonstrates the use of SERS combined with PCA and PLS to detect aromatic compounds, serum proteins, and other biomarkers for the effective detection of breast cancer. This fast, sensitive, label‐free, and non‐invasive SERS method, combined with machine learning detection, could be a powerful tool for the clinical diagnosis of breast cancer.

#### 3.4.2 Prostate Cancer (PCa)

PCa is listed by the International Agency for Research on Cancer as the second most common male‐specific disease worldwide. In the United States and Canada, PCa is a major type of cancer in males,^[^ ^203^ ^]^ and existing research indicates that Raman spectroscopy can distinguish between PCa and normal cells.^[^ ^204^ ^]^

**Figure 15:**
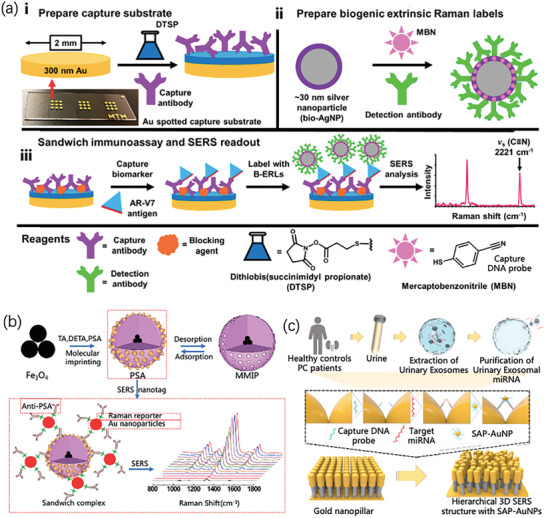
a) Schematic of the immune analysis process, sandwich immune analysis, and SERS in vitro detection of AR‐V7 protein in serum.^[^ ^235^ ^]^ Copyright 2022, American Chemical Society. b) Preparation process of the plasma sensor, schematic of PSA detection, and detected Raman spectrum.^[^ ^224^ ^]^ Copyright 2023, Elsevier. c) Schematic of the miRNA detection in urine using 3D SERS.^[^ ^234^ ^]^ Copyright 2023, Elsevier.

Changes in blood PSA levels can guide cancer diagnosis. Current screening methods for PSA in serum include electrochemical immunoadsorption,^[^ ^205^, ^206^ ^]^ optical immunoadsorption,^[^ ^207^ ^]^ fluorescence immunoadsorption,^[^ ^208^ ^]^ and SERS.^[^ ^209^, ^210^ ^]^ In particular, SERS is the most promising method and anti‐interference treatment before spectral analysis of the samples is conducive to the detection results. Molecularly imprinted polymers (MIPs) have been widely used for protein detection and analysis owing to their high selectivity, high stability, simple preparation, low cost, and high regeneration capacity.^[^ ^211^, ^212^ ^]^ Moreover, MIPs can be used in immunoassays without antibodies,^[^ ^213^, ^214^ ^]^ of which surface imprinting is the most common.^[^ ^215^, ^216^ ^]^ Imprinted polydopamine (PDA) is a simple and versatile method.^[^ ^217^, ^218^ ^]^ Turan,^[^ ^219^ ^]^ proposed SERS combined with a molecularly imprinted polymer method, and a new method for detecting PSA in serum, which forms a plasma structure between MIP and SERS. **Figure** **15**b presents the manufacturing principle of plasma sensors that can effectively detect prostate‐cancer markers in clinical practice.

Recently, liquid biopsy has been extensively used for early detection.^[^ ^220^, ^221^, ^222^, ^223^ ^]^ Similar to breast cancer, PCa can be diagnosed by detecting miRNAs in the urine.^[^ ^224^, ^225^, ^226^, ^227^ ^]^ The miRNA‐145‐5p was identified as an oncogene in PCa.^[^ ^228^ ^]^ In 2019, miRNA‐141 was reported as a diagnostic marker for PCa.^[^ ^229^ ^]^ Subsequently, Wang et al.^[^ ^230^ ^]^ designed and analyzed a novel fluorescent biosensor that promoted the clinical diagnosis of PCa. Several studies have demonstrated that Mir‐10a‐5p, Mir‐29b‐3p, mirNA‐375, miRNA‐574‐3p, and Mir‐3182 can be used as biomarkers for clinical detection.^[^ ^231^, ^232^, ^233^ ^]^ Furthermore, SERS can provide specific fingerprint spectra for target substances on rough metal nanostructures. Kim et al.^[^ ^234^ ^]^ proposed a novel method for quantitative SERS based on a 3D plasma nanostructure combined with an unlabeled miRNA‐sensing platform to detect miRNAs in urine.

Several studies have revealed that exosomes play a critical role in clinical diagnosis. Figure 15c presents the 3D hot spots formed between the Au NPs and NP heads. Ar‐v7 protein in serum is a marker for prostate detection. First, the probe DNA was fixed on the Au nanobase, and the extracted and purified miRNA and SAP‐AU NPs were combined with the substrate. The structure of 3D SERS significantly enhanced the Raman signal. Rajput et al.^[^ ^235^ ^]^ proposed a method that involved the use of quantitative NPs to enhance sandwich antibody detection and successfully detected castration‐resistant PCa in vitro. Figure 15a shows a schematic representation of AR‐V7 protein detection in patients with MCPc. Accordingly, (i) represents the substrate placed under a standard microscope, (ii) represents the preparation process of the biological exogenous Raman label, and (iii) represents the detection process and Raman spectrum.

Similar to Section 3.2.2.1, this section details the recent application of Raman spectroscopy to prostate cancer studies.

## 4 Conclusion and Outlook

**Figure 16:**
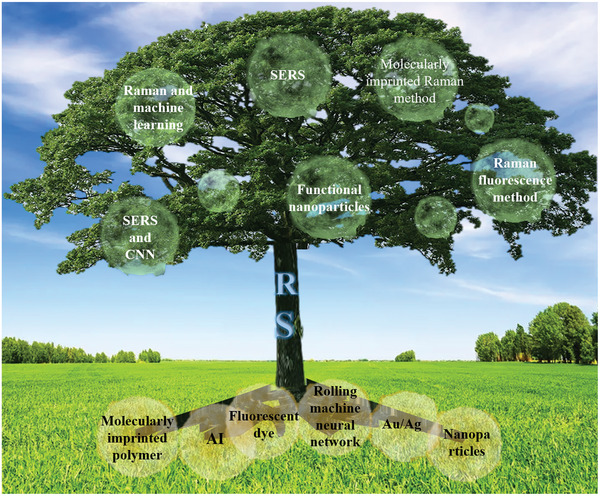
Clinical applications of Raman spectroscopic technology.

This study provides an overview of the prospects and advantages of Raman spectroscopy for the diagnosis and treatment of clinical diseases (**Figure** **16**). Additionally, we summarized the various applications of Raman spectroscopy in clinical diseases over the past 3 years (see Table 2). Finally, the status and future trends of Raman spectroscopy in clinical applications are discussed. Numerous experimental studies have shown that Raman spectroscopy can be used to distinguish patients with diseases from healthy volunteers in clinical settings. However, there is no current unified experimental standard, and the variability in experiments may lead to spectral differences among the same disease biomarkers. Therefore, optimization and standardization of experimental settings, in addition to the stability and reproducibility of experimental results, are required to promote the implementation of Raman spectroscopy technology in clinical practice.

1. Raman spectroscopy is commonly combined with multivariate analysis to obtain richer Raman information than that obtained by traditional Raman spectroscopy. However, most clinicians find it difficult and time‐consuming to process vast amounts of data; hence, Raman spectroscopy is rarely used in clinical applications. The introduction of machine learning efficiently solves this problem, and the combination of Raman spectroscopy with machine learning has potential for future research.
2. SERS NPs discovered in recent years have exhibited effective imaging in clinical applications and can be used for the diagnosis of in situ and metastatic tumors. These NPs can be implemented in prospective applications for the clinical diagnosis of deep‐site diseases. However, the application of SERS NPs in clinical image detection has led to high hardware requirements, thereby limiting their extensive use. Furthermore, the safety aspects of SERS NPs used in vivo are not fully understood, which limits their clinical application.
3. Despite the availability of portable and inexpensive Raman probes, the cost and complexity of laser sources continue to be major barriers to their widespread use in clinical translation.
4. Low‐cost, durable, firm, and high‐performance fiber‐optic probes (or equivalent light delivery devices) are needed to realize the goal of clinical translation. The transition from a research instrument to a clinically relevant device depends on the consistency and reliability of the sample and system interface. Many of these clinical translational components are now undergoing preliminary development, but more must be done to ease movement from the bench to the bedside.
5. SERS is dependent on the metal surface but is also constrained by the contact surface of the clinic. Most clinical trials have used a laser with a wavelength of 785 nm to stimulate light scattering and gather Raman signals, demonstrating its effectiveness and safety. However, lasers can only penetrate distances of less than 10 mm in real life. SERS can only be used to create metallic surfaces and samples that are sufficiently close to each other both in vitro and ex vivo.
6. In contrast to regular Raman spectroscopy, SERS exhibits a nonlinear relationship between analyte intensity and concentration owing to factors such as non‐reproducible SERS substrates and saturation effects. It is generally desirable to fabricate consistent, delicate, and clean SERS‐active substrates or introduce novel internal standards.

The applications of the different types of Raman spectroscopy vary. Based on recent studies conducted on different types of Raman spectra in clinical applications, SERS is the most commonly employed. Other types of Raman spectroscopy also demonstrate significant potential for clinical applications. It is important to emphasize that SORS has not yet been developed as a useful tool in biomedicine and has only been reported in a few studies on mouse models. Generally, clinical environments and conditions are complex and diverse. In future research, different types of Raman spectroscopy and combinations of Raman spectroscopy and other technologies should be investigated to adapt to complex and dynamic clinical environments.

Although Raman spectroscopy has demonstrated excellent potential for clinical applications, several challenges impede its clinical application. The scientific community has recognized that Raman spectroscopy faces several problems, including weak signals, long acquisition times, fluorescence interference in biological samples, time‐consuming data processing, and high cost.^[^ ^236^ ^]^ However, significant advances have been made over the past few decades with continuous advances in instrumentation technology and machine learning to address these challenges. To overcome the signal weakness and improve the SNR, complementary techniques such as SERS, RRS, and SRS have been widely used, which provide strong support for Raman spectroscopy. In the clinical field, we expect to see widespread application and further development of Raman spectroscopy techniques in the future.^[^ ^237^ ^]^ However, to realize the clinical application of Raman spectroscopy, interdisciplinary collaboration is required, with close collaboration between physicians, materials scientists, biomedical engineers, and spectroscopists to address the challenges. This will help ensure that Raman spectroscopy can be successfully applied in clinical practice, leading to further innovations and improvements in healthcare.

## Conflict of Interest

The authors declare no conflict of interest.
